# The nucleoid occlusion protein SlmA is a direct transcriptional activator of chitobiose utilization in *Vibrio cholerae*

**DOI:** 10.1371/journal.pgen.1006877

**Published:** 2017-07-06

**Authors:** Catherine A. Klancher, Chelsea A. Hayes, Ankur B. Dalia

**Affiliations:** Department of Biology, Indiana University, Bloomington, IN, United States of America; University of Geneva Medical School, SWITZERLAND

## Abstract

Chitin utilization by the cholera pathogen *Vibrio cholerae* is required for its persistence and evolution via horizontal gene transfer in the marine environment. Genes involved in the uptake and catabolism of the chitin disaccharide chitobiose are encoded by the *chb* operon. The orphan sensor kinase ChiS is critical for regulation of this locus, however, the mechanisms downstream of ChiS activation that result in expression of the *chb* operon are poorly understood. Using an unbiased transposon mutant screen, we uncover that the nucleoid occlusion protein SlmA is a regulator of the *chb* operon. SlmA has not previously been implicated in gene regulation. Also, SlmA is a member of the TetR family of proteins, which are generally transcriptional repressors. *In vitro*, we find that SlmA binds directly to the *chb* operon promoter, and *in vivo*, we show that this interaction is required for transcriptional activation of this locus and for chitobiose utilization. Using point mutations that disrupt distinct functions of SlmA, we find that DNA-binding, but not nucleoid occlusion, is critical for transcriptional activation. This study identifies a novel role for SlmA as a transcriptional regulator in *V*. *cholerae* in addition to its established role as a cell division licensing factor.

## Author summary

The cholera pathogen *Vibrio cholerae* is a natural resident of the aquatic environment and causes disease when ingested in the form of contaminated food or drinking water. In the aquatic environment, the shells of marine zooplankton, which are primarily composed of chitin, serve as an important food source for this pathogen. The genes required for the utilization of chitin are tightly regulated in *V*. *cholerae*, however, the exact mechanism underlying this regulation is currently unclear. Here, we uncover that a protein involved in regulating cell division is also important for regulating the genes involved in chitin utilization. This is a newly identified property for this cell division protein and the significance of a common regulator for these two disparate activities remains to be understood.

## Introduction

*V*. *cholerae*, the bacterium responsible for the diarrheal illness cholera, is naturally found in the marine environment. In this niche, this pathogen forms biofilms on the shells of microscopic crustaceans. The shells of these organisms are primarily composed of chitin, an insoluble polymer of β-1,4 linked *N*-acetylglucosamine (GlcNAc). Chitin is the second most abundant biopolymer on the planet, and *Vibrio* species play an important role in recycling chitin in the aquatic environment [1]. The pathway for degradation and utilization of this carbon and nitrogen source is conserved among the *Vibrionaceae* [2].

In addition to promoting the survival of *V*. *cholerae* in the aquatic environment, biofilm formation on chitin is also important for promoting transmission of this pathogen to its human host. Indeed, cholera outbreaks are seasonal in endemic areas and closely associated with blooms in chitinous zooplankton [1]. Furthermore, filtration of water through sari cloth in these areas (which has an effective pore size that will eliminate chitin biofilms but cannot filter planktonic bacteria) reduces the incidence of waterborne transmission [3, 4]. Chitin oligosaccharides also induce natural competence, a mechanism of horizontal gene transfer, in *V*. *cholerae* [5, 6]. Thus, *Vibrio*-chitin interactions are important for the persistence, transmission, and evolution of this important human pathogen in its environmental reservoir.

The expression of genes required for chitin degradation, uptake, and utilization are regulated by the orphan sensor kinase ChiS [7, 8]. ChiS activity is normally repressed by chitin binding protein (CBP), the periplasmic substrate binding protein for the chitobiose ABC transporter [7]. This repression is relieved in the presence of chitin oligosaccharides, which bind to CBP. ChiS can also be activated, however, by deletion of CBP [7]. ChiS is not predicted to directly bind DNA. Without a cognate response regulator, the mechanism of gene regulation downstream of ChiS activation is poorly understood. One locus regulated by ChiS is the *chb* operon, which encodes the genes required for uptake and utilization of the chitin disaccharide chitobiose [8]. Here, we perform an unbiased screen to identify factors downstream of ChiS required for activation of the *chb* operon and characterize one newly identified transcriptional activator of this locus.

## Results

### An unbiased screen identifies SlmA as a putative activator of chitin utilization genes in *V*. *cholerae*

**Fig 1:**
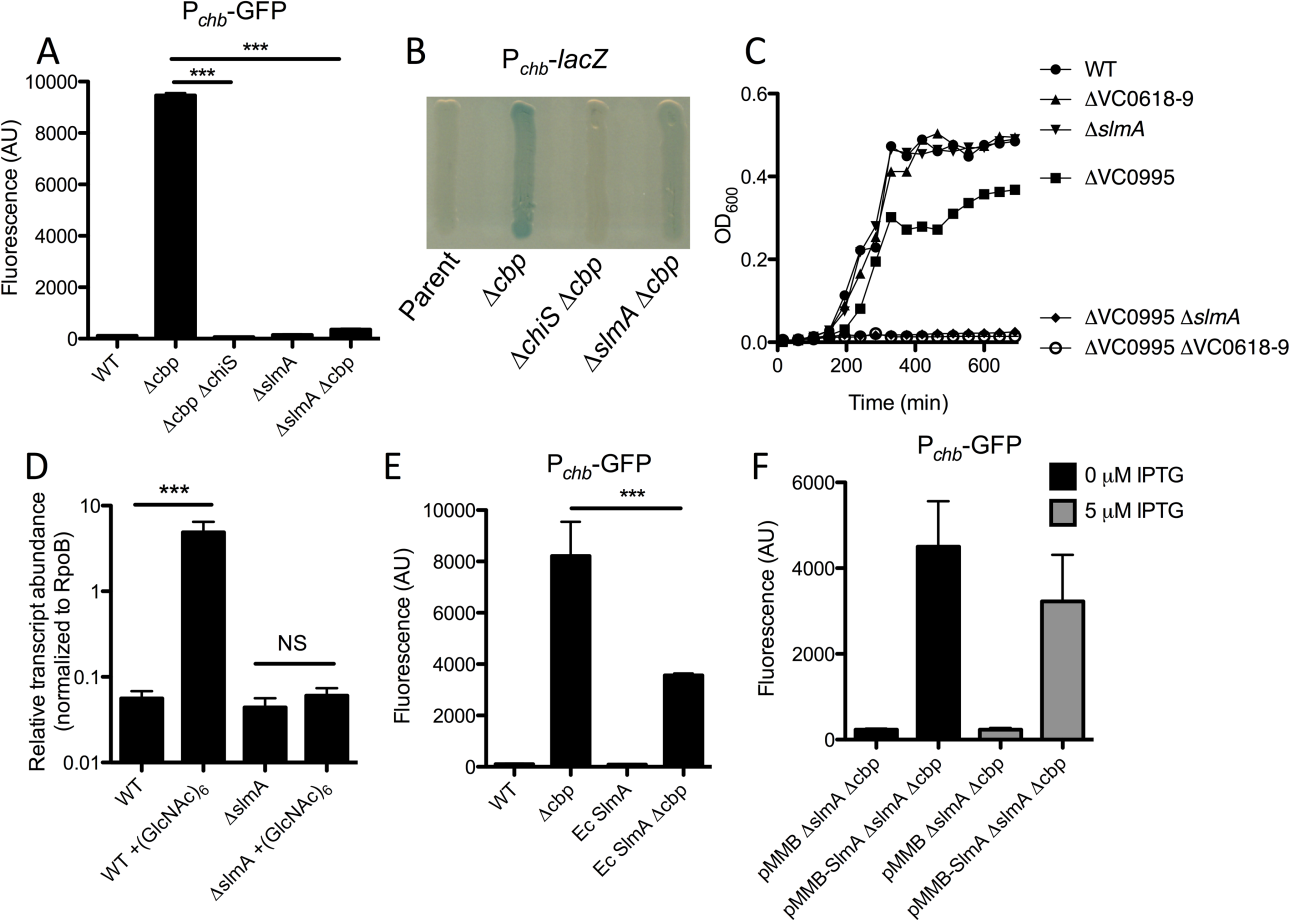
SlmA is required for transcriptional activation of P_*chb*_. (**A**) GFP fluorescence was measured in the indicated strains, all of which contain a P_*chb*_-*gfp* transcriptional reporter. Statistics shown indicate that samples are significantly different from the “Δ*cbp”* sample. (**B**) Transcription of P_*chb*_ was schematized on X-gal-containing media in the indicated strains, all of which contained a P_*chb*_-*lacZ* transcriptional reporter. (**C**) Growth curves of the indicated strains in M9 minimal medium containing 0.5% chitobiose as a sole carbon source. (**D**) qRT-PCR of the *chb* short transcript was measured in WT and Δ*slmA* cells grown in the presence or absence of (GlcNAc)_6_. (**E**) GFP fluorescence was measured in the indicated strains, all of which contain a P_*chb*_-*gfp* transcriptional reporter. (**F**) GFP fluorescence was measured in a P_*chb*_-*gfp* Δ*slmA* Δ*cbp* strain harboring either an empty vector (pMMB) or a SlmA expression vector (pMMB-SlmA) grown in the presence or absence of 5 μM IPTG. Data in **A**, **D**, **E**, and **F** are shown as the mean ± SD and are from at least three independent biological replicates. Data from **B** and **C** are representative of at least 2 independent experiments. *** = *p*<0.001, NS = not significant.

To study ChiS regulation of the *chb* operon, we first generated a P_*chb*_ transcriptional reporter. As expected, this reporter was activated in a *cbp* mutant, and this activation was dependent on ChiS (**Fig 1A**). We exploited this fact and used our transcriptional reporter to perform two independent transposon mutant screens to identify potential activators and repressors of this locus. To identify repressors, we used a P_*chb*_-*lacZ* reporter (which forms white colonies), and screened for mutant blue colonies. Conversely, we screened for activators by using a P_*chb*_-*lacZ* Δ*cbp* strain (which forms blue colonies) and isolated mutant white colonies. A positive control for the repressor screen was *cbp*, while a positive control for the activator screen was *chiS*. As expected, both of these genes were identified in their respective screens, which helped to validate this approach. No additional hits were identified in the repressor screen, but one hit that we identified in the activator screen was VC0214, the gene encoding the nucleoid occlusion protein SlmA (**Fig 1A**). Consistent with a role in transcriptional activation of this locus, P_*chb*_ is no longer induced in a Δ*slmA* Δ*cbp* mutant strain (**Fig 1A** and **1B**). To determine if SlmA is also required for activation of this locus under more physiologically relevant conditions, we assessed *cbp* transcript levels in WT and Δ*slmA* cells when induced with (GlcNAc)_6_, the chitin hexasaccharide. While this locus is strongly induced in the WT, there is little to no activation in the *slmA* mutant, which is consistent with what we observed when using a *cbp* mutant to artificially activate this locus (**Fig 1D**). Using 5’ RACE, we mapped the transcription start sites (TSS) for the *chb* transcript and found that there are two distinct TSSs for this locus. One TSS representing a longer transcript that is basally expressed in the absence of chitin induction (uninduced transcript), and a second TSS for a shorter transcript that is expressed when this locus is induced by chitin oligosaccharides or artificially induced by deletion of *cbp* (induced transcript) (**S1 Fig**). Using primers that are specific to the longer uninduced transcript, we find that levels of this transcript are induced only 2 to 4-fold when cells are grown in the presence or absence of (GlcNAc)_6_ in both WT and Δ*slmA* cells, indicating that SlmA is specifically playing a role in the activation of this locus at the downstream TSS (**S1 Fig**).

The *chb* operon encodes an ABC transporter that mediates uptake of chitobiose (permease encoded by VC0618-0619). *V*. *cholerae*, however, can also degrade chitobiose in the periplasm into the chitin monomer GlcNAc [2, 9, 10], which is taken up by a PTS transporter, VC0995, that is outside of the P_*chb*_ operon and whose regulation is independent of ChiS (**S2 Fig**) [8]. Thus, deletion of either transporter independently does not eliminate growth on chitobiose (**Fig 1C**). However, upon deletion of both the chitobiose and GlcNAc transporters, cells are unable to grow on chitobiose as the sole carbon source (**Fig 1C**). To determine if SlmA-dependent gene regulation of P_*chb*_ was physiologically relevant, we assessed whether SlmA was required for growth on chitobiose ((GlcNAc)_2_) as a sole carbon source. When we performed this analysis, we found that SlmA was required for growth on chitobiose in a ΔVC0995 mutant background, which is consistent with SlmA playing a physiologically important role in regulating the chitobiose transporter in the *chb* operon. As expected, all strains grew equally well when glucose or tryptone was provided as the sole carbon source whereas any strains with a ΔVC0995 mutation were unable to grow on GlcNAc (**S2 Fig**). A *slmA* mutant, however, does not phenocopy a *chiS* mutant. In fact, a Δ*chiS* strain is unable to grow on (GlcNAc)_2_ as the sole carbon source, which is not surprising since this regulator is required for the expression of a number of chitin catabolic genes in addition to the *chb* operon [8](**S3 Fig**).

SlmA function has primarily been characterized in *E*. *coli* [11–15]. This protein, however, is well conserved between *E*. *coli* and *V*. *cholerae* (67% identity, 83% similarity; **S4 Fig**). To determine if gene regulation by SlmA is a conserved property, we replaced the native copy of *slmA* in *V*. *cholerae* with the gene for *slmA* from *E*. *coli*. We found that *Ec* SlmA was able to activate transcription of P_*chb*_, albeit, less effectively than *Vc* SlmA, suggesting that this activity is not unique to the *Vc* homolog, but is a conserved property of this protein (**Fig 1E**).

To further confirm that deletion of *slmA* was responsible for the phenotypes observed on the P_*chb*_ operon, we complemented *slmA* on a plasmid. As expected, we found that ectopic expression of SlmA was sufficient to recover activation of P_*chb*_ in a Δ*slmA* Δ*cbp* mutant (**Fig 1F**). As seen previously [16], expression from the pMMB vector used here is leaky and as a result, basal expression of SlmA from this plasmid without inducer is sufficient to activate P_*chb*_ (**Fig 1F**).

### SlmA is a direct transcriptional activator of P_*chb*_

**Fig 2:**
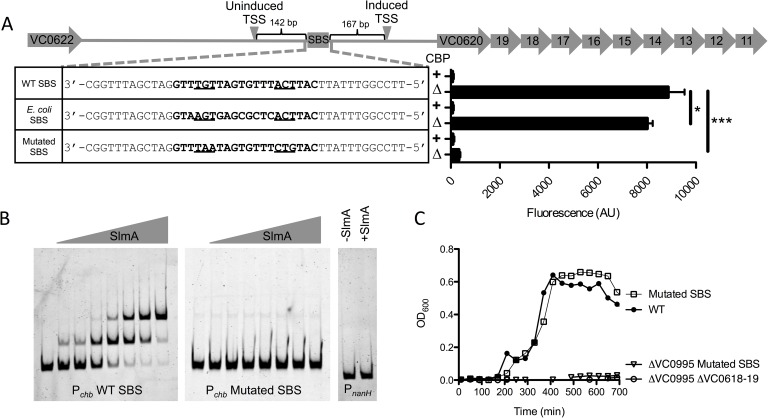
SlmA is a direct transcriptional activator of P_*chb*_. (**A**) Promoter map of P_*chb*_ showing the SBS and the TSSs mapped in the presence (induced) and absence (uninduced) of chitin. “WT SBS” represents the native P_*chb*_ sequence, “*E*. *coli* SBS” indicates that the native SBS was swapped for the consensus SBS from *E*. *coli*, and “mutated SBS” indicates that the 6 highly conserved residues in the SBS were mutated. SBS sequences for the indicated mutants are shown in bold text and the 6 most highly conserved residues of the SBS are underlined. GFP fluorescence was measured in strains harboring the indicated mutations in the P_*chb*_-*gfp* transcriptional reporter. The *cbp* status of each strain is indicated by a “+” (*cbp* intact) or a “Δ” (Δ*cbp* strain background). Data are shown as the mean ± SD and are from at least three independent biological replicates. (**B**) EMSAs performed with purified SlmA and the indicated promoter probes. The two P_*chb*_ probes were incubated with (from left to right) 0 nM, 7.5 nM, 15 nM, 30 nM, 60 nM, 120 nM, 240 nM, or 480 nM SlmA. The P_*nanH*_ probe was incubated with 0 nM or 480 nM SlmA. (**C**) Growth curves of the indicated strains in M9 minimal medium containing 0.5% chitobiose as the sole carbon source. Data from **B** and **C** are representative of at least 2 independent experiments. *** = *p*<0.001, * = p<0.05, NS = not significant.

SlmA is a TetR family protein and binds to a specific and well-defined sequence [12]. Through bioinformatic analysis, we identified one SlmA binding site (SBS) in P_*chb*_ based on the SBS consensus sequence of *E*. *coli* SlmA (**Fig 2A**) [12]. This was further confirmed by a recent *in vitro* whole genome binding analysis that identified putative SBSs in *V*. *cholerae* [17].

To determine if SlmA could bind to this site, we performed electrophoretic mobility shift assays (EMSAs) using a probe from P_*chb*_ that contains the putative SBS. Indeed, we found that SlmA could directly bind to P_*chb*_ (**Fig 2B**). SlmA was previously shown to bind to an SBS as a dimer-of-dimers [14], which is consistent with the two shifts we observed with the P_*chb*_ promoter probe (**Fig 2B**). As a negative control, we tested binding of SlmA to P_*nanH*_, an unrelated promoter that lacks a predicted SBS. As expected, we found that SlmA could not bind to this probe (**Fig 2B**). To determine if the putative SBS in P_*chb*_ is responsible for the shift observed in our EMSAs, we mutated 6 highly conserved residues in the SBS. We found that SlmA no longer shifted the P_*chb*_ probe when these residues were mutated, suggesting that this sequence represents a *bona fide* SBS in the *chb* promoter (**Fig 2B**).

Since we found that SlmA could directly bind to an SBS in the *chb* promoter, we next wanted to determine if this site was important for the transcriptional activation of this locus. To that end, we assessed activation of P_*chb*_ in a mutant strain where we mutated the SBS. When the SBS was mutated, we no longer observed activation of P_*chb*_ (**Fig 2A**). Promoter truncations of P_*chb*_ also confirmed that the SBS in this promoter was required for activation of this locus (**S5 Fig**). Furthermore, we replaced the native SBS in the P_*chb*_ promoter with the consensus SBS for *E*. *coli* SlmA. This alternative SBS was able to support activation of the locus (**Fig 2A**). Cumulatively, these results suggest that SlmA recruitment to the P_*chb*_ promoter via an SBS is required for transcriptional activation. Our previous results indicated that *Ec* SlmA supported activation of P_*chb*_ less effectively than *Vc* SlmA (**Fig 1E**). To determine if this was due to a reduced affinity of *Ec* SlmA for the SBS in P_*chb*_, we tested activation of P_*chb*_ containing the consensus *Ec* SBS in a strain expressing *Ec* SlmA at the native locus. This strain, however, had similar levels of P_*chb*_ induction compared to a strain with *Ec* SlmA and the native SBS at P_*chb*_ (**S6 Fig**). Thus, reduced activation of *Ec* SlmA is likely due to a reduced ability to promote transcriptional activation and not due to reduced affinity for the SBS.

Above, we showed that SlmA was required for regulation of the chitobiose ABC transporter encoded by the *chb* operon for optimal growth on chitobiose (**Fig 1C**). Deletion of SlmA, however, may have pleiotropic effects. To determine if SlmA binding at the SBS in P_*chb*_ was responsible for this effect, we mutated the SBS in the native *chb* promoter and tested growth on chitobiose. We found that mutating the SBS in P_*chb*_ prevents growth on chitobiose in the ΔVC0995 background, which is consistent with this SBS being critical for regulating the chitobiose transporter in the *chb* operon (**Fig 2C**). These results indicate that SlmA-dependent regulation of P_*chb*_ is both direct and physiologically relevant.

### DNA binding, but not nucleoid occlusion activity, is required for SlmA-dependent transcriptional activation of P_*chb*_

**Fig 3:**
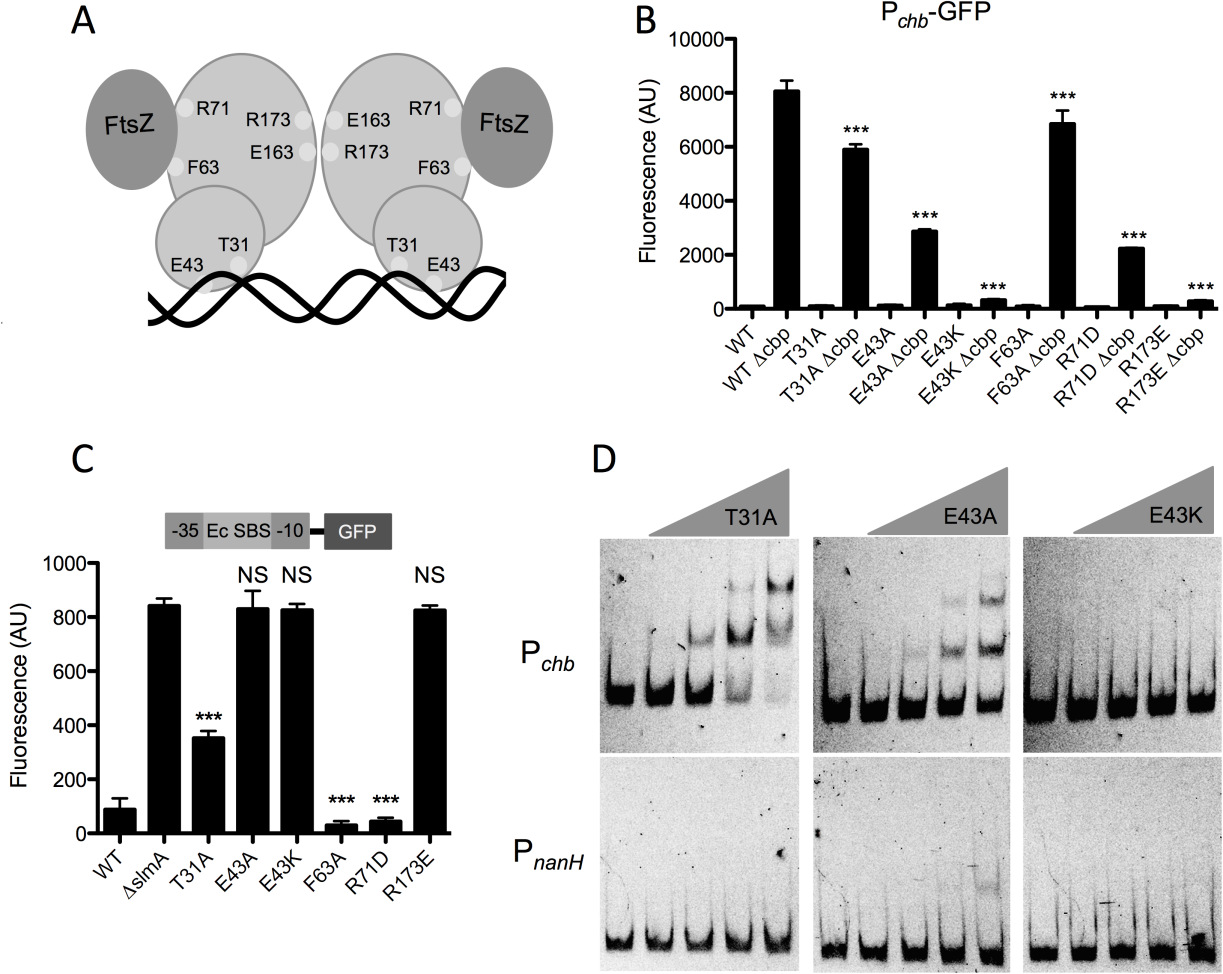
DNA binding, but not nucleoid occlusion activity, is required for SlmA-dependent transcriptional activation of P_*chb*_. (**A**) Schematic of SlmA depicting residues mutated in this study. T31 and E43 are predicted to interact with DNA, F63 and R71 mediate FtsZ interactions, and R173 is required for dimerization. (**B**) GFP fluorescence was measured in strains containing a P_*chb*_-*gfp* reporter and the indicated site-directed mutations in the native copy of *slmA*. Statistical comparisons are made to the “WT Δ*cbp”* sample. (**C**) GFP fluorescence was assessed in strains harboring an SBS-*gfp* reporter that is a readout for DNA binding and contain the indicated site-directed mutations in the native copy of *slmA*. Statistical comparisons are made to the “Δ*slmA”* sample. (**D**) EMSAs performed with the WT P_*chb*_ probe or the negative control P_*nanH*_ probe incubated with (from left to right): 0 nM, 240 nM, 480 nM, 960 nM, or 1920 nM of the indicated SlmA mutant protein. Data in **B** and **C** are shown as the mean ± SD and are from at least three independent biological replicates. Data from **D** is representative of at least two independent experiments. *** = *p*<0.001, NS = not significant.

The ability of SlmA to mediate nucleoid occlusion is dependent upon its ability to dimerize, bind DNA, and interact with FtsZ. The residues involved in these interactions have been previously characterized [11–14]. We next wanted to dissect which functional interactions of SlmA are required for transcriptional activation of P_*chb*_. So, we mutated residues in SlmA involved in DNA binding (T31, E43), FtsZ interaction (F63, R71), and dimerization (R173) (**Fig 3A**). We then tested these mutants for their ability to activate expression of P_*chb*_. We also assessed whether these SlmA variants could bind to DNA using a previously described synthetic SBS-*gfp* reporter where SlmA binding at a high affinity SBS represses expression of GFP [11]. We used the consensus SBS from *E*. *coli* instead of the native SBS found in P_*chb*_ because SlmA binds to this SBS with a higher affinity, allowing for a more sensitive evaluation of DNA binding (**S7 Fig**). We also tested the nucleoid occlusion activity of these SlmA variants by overexpressing each and assessing the morphology of cells. Overexpression of WT SlmA results in a dramatic filamentous phenotype due to excess nucleoid occlusion activity, whereas alleles deficient in nucleoid occlusion do not cause this phenotype [15](**S9 Fig**). We also triple-FLAG tagged all SlmA alleles tested at the native locus to assess their expression levels by western blot analysis. This analysis demonstrated that all mutant alleles were expressed at least at WT levels (**S8 Fig**). Importantly, SlmA-triple FLAG constructs were still functional for P_*chb*_ activation (**S8 Fig**).

First, we found that the FtsZ binding mutants (F63A and R71D) were still able to facilitate transcriptional activation of P_*chb*_, although one mutant (R71D) displayed a reduced degree of activation (**Fig 3B**). As expected, these SlmA variants were still able to bind DNA (**Fig 3C**) and had lost nucleoid occlusion activity (**S9 Fig**). These results suggest that the nucleoid occlusion activity of SlmA is not required for its ability to act as a transcriptional activator of the *chb* operon.

Next, we tested DNA binding mutants T31A and E43A that were previously identified in *E*. *coli* [11], and, surprisingly, we found that these variants were still able to activate transcription of P_*chb*_. The T31A mutant was still able to bind DNA as indicated by the synthetic SBS-GFP reporter (**Fig 3C**). The E43A mutant, however, was not able to bind DNA in this assay (**Fig 3C**). Our previous results with the mutated SBS would suggest that SlmA binding to P_*chb*_ is required for activation, therefore, we hypothesized that the E43A mutant may still have the ability to bind an SBS, however, this may be below the limit of detection of our DNA binding reporter. To test this, we purified SlmA E43A and assessed its ability to bind P_*chb*_ in an EMSA. Indeed, we found that SlmA E43A was still able to bind to this promoter, although with a greatly reduced affinity compared to WT SlmA or SlmA T31A (**Figs 3D** and **2B**). Thus, these results indicate that while these mutations may eliminate DNA binding of *Ec* SlmA [11], they may have a limited or reduced impact on DNA-binding of *Vc* SlmA. Overexpression of these DNA binding mutants indicated that they had lost the ability to mediate nucleoid occlusion (**S9 Fig**).

Dimerization of SlmA is required for DNA binding as well as nucleoid occlusion [11]. Dimerization requires a charge-based interaction of R173 from one monomer with E163 of the other monomer. Swapping the charge of either residue can prevent dimerization [11], thus we used an R173E mutant to test the role of SlmA dimerization. The dimerization mutant R173E was unable to activate P_*chb*_. As expected, this variant had also lost the ability to bind DNA (**Fig 3C**) and mediate nucleoid occlusion (**S9 Fig**).

Next, we sought to identify residues that are required for SlmA-mediated activation of P_*chb*_. To that end, we performed error-prone PCR of SlmA and screened for colonies that had lost the ability to activate P_*chb*_-*lacZ*. We then counter-screened colonies for the ability to bind DNA using our synthetic SBS-GFP reporter. We screened >15,000 colonies and identified ~500 colonies that were deficient for activation of P_*chb*_-*lacZ*. In our counter-screen, however, we found that none of these SlmA mutants maintained the ability to bind DNA, suggesting that DNA binding and transcriptional activation of P_*chb*_ are tightly linked. One variant identified in this screen was SlmA E43K, which is the same residue previously identified as playing a role in DNA binding [14], however, with a lysine substitution in place of alanine. We hypothesized that the E43K mutant could no longer bind to DNA, in contrast to the E43A variant studied above. To test this, we purified SlmA E43K and assessed its ability to bind P_*chb*_ *in vitro*. Indeed, by EMSA we found that E43K could not bind P_*chb*_ (**Fig 3D**). Importantly, this mutation does not simply result in an unstable protein because the E43K mutant still makes WT levels of SlmA protein (**S8 Fig**). This mutant also lost the ability to mediate nucleoid occlusion when overexpressed (**S9 Fig**). Cumulatively, these results indicate that high affinity binding of SlmA to DNA may be required for proper nucleoid occlusion, while weak binding is sufficient to mediate transcriptional activation of P_*chb*_.

### SlmA is not sufficient to promote activation of P_*chb*_

Thus far, our data suggest that activation of P_*chb*_ requires activation of ChiS, either via deletion of *cbp* or induction with chitin oligosaccharides. To determine if SlmA is the sole activator that acts downstream of ChiS we decided to overexpress SlmA to see if it was sufficient to activate P_*chb*_ in the absence of either inducer. Overexpression of WT SlmA results in a dramatic filamentous phenotype (**S9 Fig**). Interestingly, overexpression of WT SlmA from a chromosomally integrated P_*tac*_-SlmA construct still induced P_*chb*_ expression in a Δ*cbp*-dependent manner, even at levels that induced morphological defects (10 μM), although activation was lost when cultures were induced with 100 μM IPTG (**S10 Fig**). Above, we show that the nucleoid occlusion mutants T31A, F63A, and R71D are still able to activate P_*chb*_ (**Fig 3B**), while their overexpression does not result in filamentation (**S9 Fig**). Indeed, overexpression of SlmA F63A from a chromosomally integrated P_*tac*_-SlmA F63A construct still supported activation in a Δ*cbp*-dependent manner, however, induction with 100 μM IPTG still prevented activation similar to what was observed with overexpression of WT SlmA (**S10 Fig**). Also, this was independent of any obvious morphological defect, suggesting that overexpression of SlmA at this level prevents P_*chb*_ activation independent of its role in nucleoid occlusion (**S10 Fig**).

Next, to determine if SlmA was sufficient to mediate P_*chb*_ activation, we overexpressed SlmA T31A, F63A, and R71D mutants with 10 μM IPTG and assessed whether we could activate P_*chb*_ under non-inducing conditions (i.e. in a *cbp*^+^ strain grown in the absence of chitin oligosaccharides). Overexpression of these constructs, however, did not result in activation under non-inducing conditions (**S11 Fig**). Cumulatively, these results indicate that the repression of ChiS by CBP plays a dominant role over the activity of SlmA as a transcriptional activator of P_*chb*_. This is consistent with a model whereby another, as yet unidentified, protein acts downstream of ChiS and is required for coactivation of this locus along with SlmA.

Since overexpression of SlmA F63A with 100 μM IPTG inhibited P_*chb*_ activation, we decided to test whether this phenotype was the result of excess SlmA protein, which titrated away a putative co-activator from the P_*chb*_ operon. To that end, we overexpressed ChiS, which might enhance the expression / activity of this putative downstream coactivator. Interestingly, ChiS overexpression at 10–1000 μM also prevented P_*chb*_ activation, albeit, not to the degree of SlmA F63A overexpression (**S10 Fig**). ChiS overexpression, however, did not rescue the loss of P_*chb*_ activation when SlmA F63A was overexpressed (**S10 Fig**). The lack of increased P_*chb*_ expression over the native levels in the presence of higher levels of ChiS and/or SlmA indicate that neither protein is limiting and that native levels of these proteins are sufficient for maximal activation of P_*chb*_.

### Sequence specificity between the SBS and induced promoter for P_*chb*_ activation suggests a possible coactivator-binding site

The transcriptional regulator cAMP receptor protein (CRP) binds to a specific DNA sequence in the presence of cAMP, allowing for activation or repression of target genes. One turn of B DNA is approximately 10 bp, and moving the CRP binding site in increments of 10 bp proximally or distally from the promoter maintains transcriptional activation of genes regulated by CRP by maintaining the helical phase of the CRP binding site in relation to the promoter [18, 19]. Conversely, moving the CRP binding site in increments of less than 10 bp places the CRP binding site out of helical phase with the promoter and therefore prevents activation by disrupting proper interaction with RNA polymerase (RNAP).

We tested whether there was helical phase-dependence for SlmA-mediated activation of P_*chb*_ by a similar mechanism. We found that moving the SBS as little at 3 bp or as much as 12 bp resulted in a significant decrease in P_*chb*_ activation (**S12 Fig**). Constructs in which the SBS was moved to maintain the helical phase (i.e. in increments of 10 bp) were also unable to promote robust activation, indicating that SlmA may not activate transcription by direct contact with RNA polymerase in a mechanism similar to CRP (**S12 Fig**). This is in line with the distance of the SBS from the TSS, 167 bp, which makes it unlikely that SlmA bound to its SBS directly interacts with RNAP to activate transcription. This supports the hypothesis that a coactivator may promote SlmA-dependent activation of P_*chb*_.

**Fig 4:**
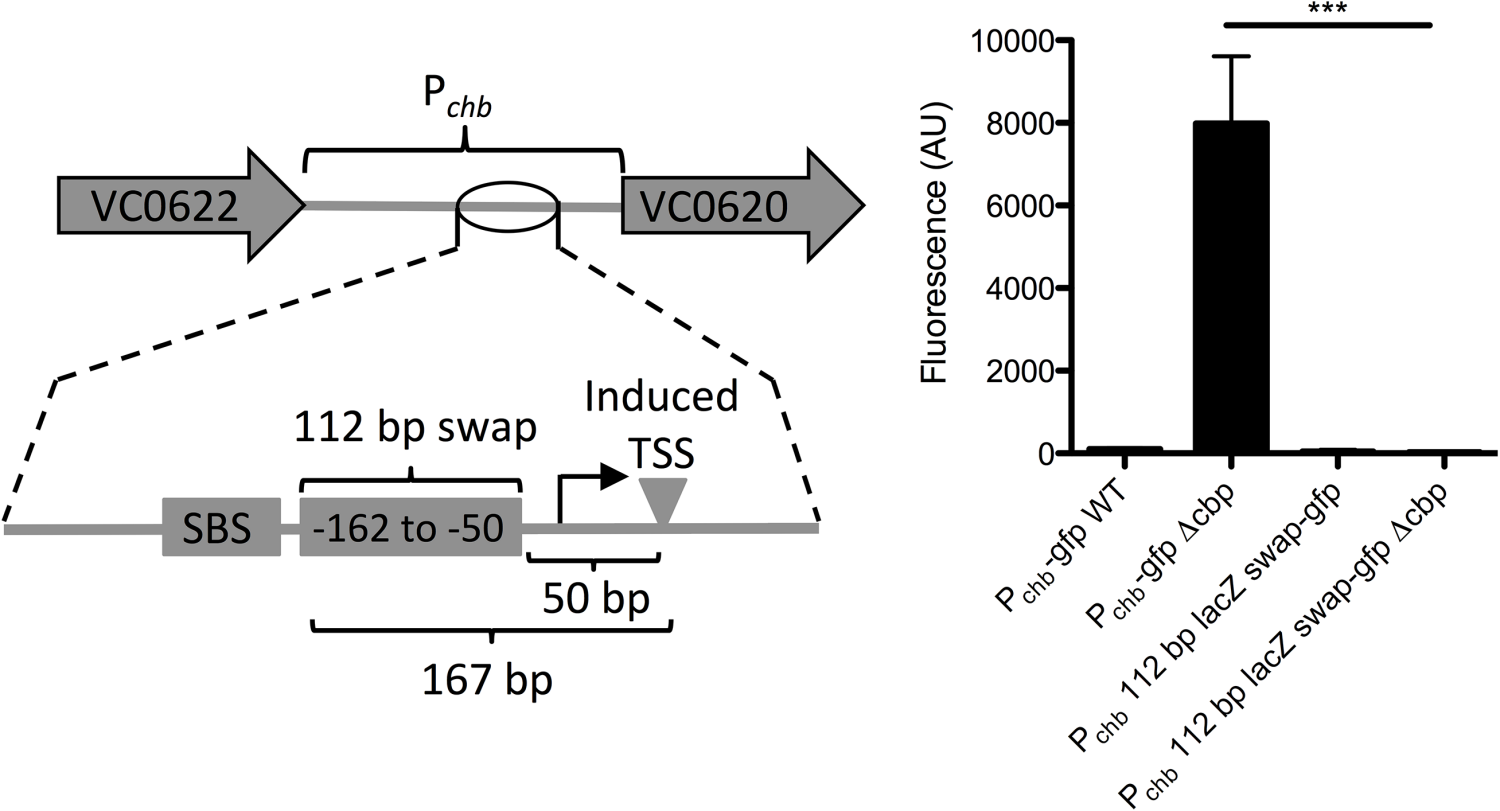
Sequence specificity between the SBS and induced promoter for P_*chb*_ activation. A 112 bp fragment of P_*chb*_-*gfp* (from -162 to -50 bp) was swapped out with a fragment of equal size from the *lacZ* gene and activation was determined by assessing fluorescence. All data are shown as the mean ± SD and are from at least three independent biological replicates. *** = *p*<0.001.

Next, we assessed whether there was sequence specificity for activation of P_*chb*_ in the region between the SBS and promoter element for the chitin-induced TSS. We hypothesized that if a coactivator was required for expression, there may be sequence specificity within this region, while if no coactivator was required, maintaining the spacing between the SBS and TSS may be sufficient to support activation. We tested this by replacing the region between the SBS and the -35 signal with the same number of nucleotides from an intragenic region of the *lacZ* gene (**Fig 4**). The activation of this reporter was abolished after swapping the native sequence with *lacZ* sequence (**Fig 4**), indicating that sequence specificity within this region may be required for activation. Furthermore, truncation studies indicated that elements necessary for activation include the SBS, TSS and intervening regions (**S5 Fig**). Also, a bioinformatically determined hairpin exhibits some inhibitory activity (**S5 Fig**). Together, these data are consistent with a model whereby the region between the SBS and TSS contains a coactivator-binding site required for P_*chb*_ induction. There are, however, a number of alternative models that can account for the results obtained, which are addressed in the **Discussion**.

Finally, we were interested in determining which sigma factor was required for activation of P_*chb*_. To test this, we inactivated every non-essential sigma factor (RpoN, RpoS, RpoE, RpoF, and RpoH) in *V*. *cholerae* and assessed transcriptional activation of P_*chb*_. We observed activation in all mutant strains, suggesting that the essential housekeeping sigma factor RpoD likely plays a dominant role in P_*chb*_ expression (**S13 Fig**). The degree of P_*chb*_ activation, however, was reduced in the *rpoE* mutant, suggesting that this sigma factor either plays a minor role in activation of this locus or that RpoE is required for proper expression or folding of the GFP reporter. Additionally, we observed an increase in expression in the *rpoS* mutant, suggesting that this sigma factor may play a role in the expression of some upstream repressor of this locus.

## Discussion

Here, we show that the cell division licensing factor SlmA is required for activation of chitobiose utilization genes in *V*. *cholerae*. We show that this regulation is direct because SlmA binds to P_*chb*_, the promoter that regulates these genes. Furthermore, we demonstrate that this direct regulation is required for growth of *V*. *cholerae* on chitobiose.

**Fig 5:**
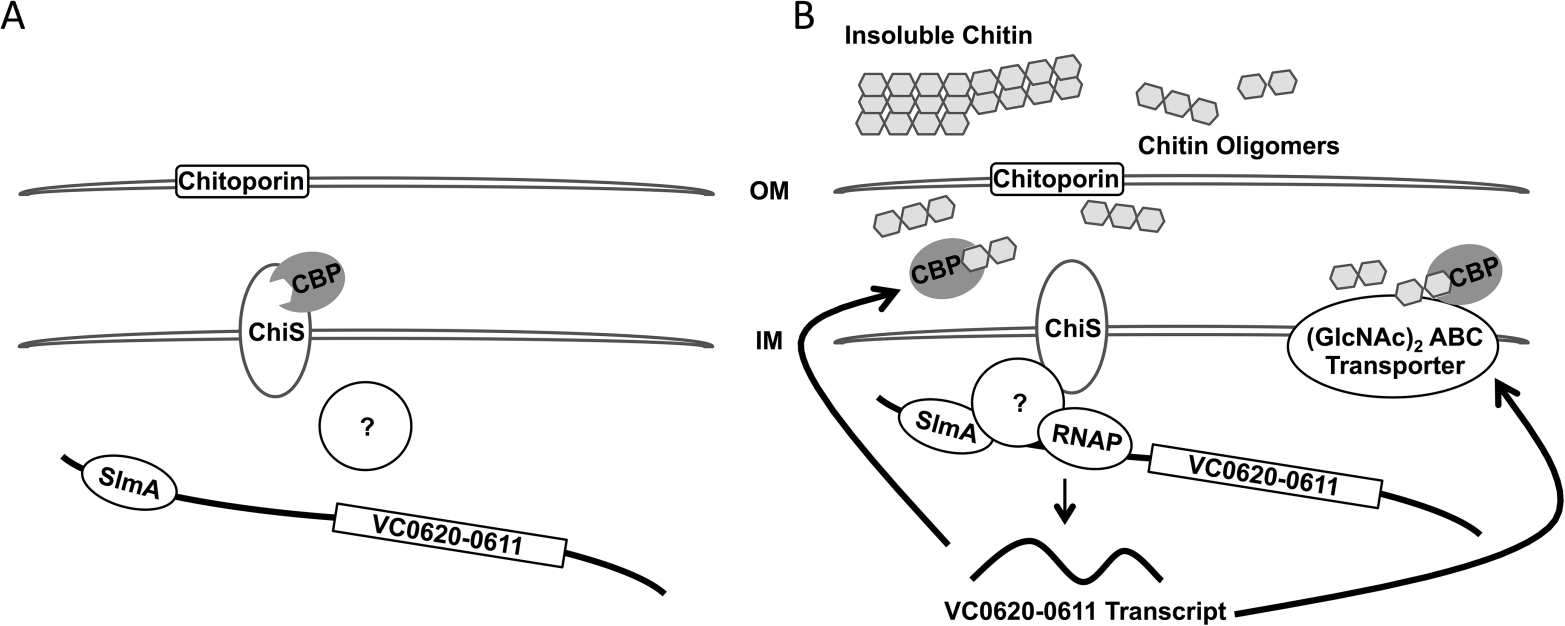
Model for transcriptional activation of P_*chb*_. (**A**) In the absence of chitin, CBP inhibits ChiS from activating expression of P_*chb*_. (**B**) In the presence of chitin, CBP binds chitin oligomers, which relieves repression of ChiS. We hypothesize that ChiS interacts with or activates another currently unidentified protein, which recruits RNA Polymerase to activate transcription of P_*chb*_ in a SlmA-dependent manner. SlmA may help to recruit the putative factor to the P_*chb*_ promoter or is an otherwise required coactivator of this locus. Upon expression of the *chb* operon, proteins involved in chitin uptake and utilization of chitobiose, including CBP and the chitobiose ABC permease, are synthesized.

While we show that SlmA is a direct activator of P_*chb*_, the exact mechanism of activation is still unclear. We have shown that the location of the SBS within P_*chb*_ is important for activation. Additionally, we show that the sequence between the SBS and the -35 sequence is required for activation, consistent with this region harboring a coactivator binding site. Because the SBS is located 167 base pairs away from the TSS, it is unlikely that SlmA directly interacts with RNA Polymerase to mediate activation. We also explored the possibility that SlmA binding may occlude a repressor binding site thereby allowing for activation of P_*chb*_ indirectly. We formally tested this hypothesis by performing a Tn mutant screen for repressors in a Δ*slmA* Δ*cbp* P_*chb*_-*lacZ* reporter strain, however, this screen did not identify any putative repressors (~70,000 mutants visually screened). As a result, we propose a mechanism in which SlmA requires a presently unknown ChiS-dependent coactivator for activation of P_*chb*_ (**Fig 5**). A candidate for this unknown factor was not identified in the Tn-mutant screen for activators that identified SlmA. This screen, however, may not have been saturating or, alternatively, this factor may be essential for viability or masked by genetic redundancy.

Because ChiS is an orphan sensor kinase, the most logical coactivator would be a cognate response regulator. However, it has been shown that the conserved histidine and aspartate residues of ChiS that are critical for autophosphorylation and phosphorelay activity, respectively, are not important for activation of P_*chb*_ [20]. Thus, while the identity of the regulator that acts downstream of ChiS remains unknown, we believe it is unlikely to be a classical response regulator. It is possible that this coactivator bridges SlmA and RNA polymerase to mediate transcriptional activation (as depicted in **Fig 5**). Alternatively, it is possible that the coactivator loops, bends, or structurally alters DNA in the *chb* promoter to enhance SlmA-dependent recruitment of RNA polymerase. Identifying and characterizing a putative coactivator for P_*chb*_ that acts downstream of ChiS will be the focus of future work.

Another possible mechanism of activation is that SlmA directly interacts with RNA polymerase through its ability to polymerize and spread on DNA [14]. We have two results, however, that diminish this as a likely model for activation. First, we show that moving the SBS as little as 3 bp in either direction within P_*chb*_ abolishes activation (**S7 Fig**). If DNA spreading were critical we would predict that this perturbation should have a limited effect on transcriptional activation. Further, we have shown that overexpression of SlmA can actually decrease P_*chb*_ transcription, which again would not be predicted by a DNA spreading model (**S10 Fig**). Alternatively, it is possible that increased SlmA spreading (when SlmA is overexpressed) may prevent RNA polymerase from binding, resulting in decreased expression of P_*chb*_.

We have shown that there are two transcriptional start sites for the *chb* locus. The short transcript is strongly induced in the presence of chitin oligosaccharides, while the long transcript is basally produced in the absence of chitin and is only modestly induced in its presence (2–4 fold). This indicates that there may be an additional level of regulation involving the 5’ untranslated region (UTR) of the *chb* transcript. A putative hairpin in P_*chb*_ also seemed to have some inhibitor effect on P_*chb*_ expression (**S5 Fig**). It is possible that the long transcript and hairpin play an important role in the basal expression of the *chb* operon, which is required for a rapid response to chitin oligosaccharides.

TetR proteins are generally transcriptional repressors, however, there are examples where these proteins can mediate transcriptional activation. One well-studied example is the LuxR protein in *Vibrio harveyi* (also known as HapR in *V*. *cholerae* and SmcR in *Vibrio vulnificus*) [21, 22]. SlmA is primarily characterized as a TetR family cell wall licensing factor and here we describe its additional role as a transcriptional activator. It has been hypothesized that transcriptional regulatory proteins arose from nucleoid-associated proteins [23]. Their role in binding DNA to properly structure the nucleoid may have evolved to aid in transcriptional regulation by contributing to the rearrangement of DNA structure at promoter regions [24]. One such example is integration host factor (IHF), which binds DNA and results in dramatic DNA bending (bend angle of ~120°), which can promote activation of regulated genes. SlmA binding results in subtle bending of DNA (~18°) [14]. Thus, SlmA may have evolved from a protein that structures the nucleoid into a cell division licensing factor as well as a transcriptional regulator. Additionally, since SlmA carries out both nucleoid occlusion and transcriptional activation, it is possible that these two activities affect one another. As a result, co-option of SlmA for regulation of P_*chb*_ may be a mechanism to integrate the cell division status of the cell with activation of chitin utilization.

To our knowledge, this is the first example of gene regulation by a nucleoid occlusion protein. A recent *in vitro* whole genome binding analysis identified 79 putative SBSs in *V*. *cholerae* [17]. We determined that ~25% (20/79) of these putative SBSs are in intergenic sites (including the one in P_*chb*_), while only ~12% of the genome constitutes intergenic sequence. By contrast, in *E*. *coli*, ~8.3% (2/24) of SBSs are in intergenic sites [12] and ~11% of the genome is intergenic. Also, analyzing binding sites for Noc, the nucleoid occlusion protein of *Bacillus subtilis*, ~12% (9/74) of binding sites are intergenic [25] and ~11% of the genome represents intergenic sequence. By contrast, for binding sites for the terminus macrodomain proteins MatP (known as *matS* sites) ~24% (6/25) are in intergenic regions [26]. Thus, it is possible that enriched binding of nucleoid-associated proteins at intergenic regions (as is the case for *matS* sites in *E*. *coli* and SBSs in *V*. *cholerae*) is because these sites are more flexible in regards to accommodating mutations compared to coding regions of the genome. Alternatively, SBSs may be enriched in intergenic sites in *V*. *cholerae* possibly to regulate the expression of additional genes. This will be the focus of future work. Preliminary RNA-seq analysis of a *slmA* mutant grown in rich medium, however, did not uncover any additional loci regulated by SlmA in *V*. *cholerae*. An effect of SlmA on gene regulation at P_*chb*_, however, was only uncovered by deletion of *cbp* or growth on chitin (both of which induce this locus). Thus, it may be that additional inducing cues are required to uncover a role for SlmA-dependent regulation at additional genetic loci. Nucleoid occlusion factors are present across diverse bacterial genera and it is tempting to speculate that these proteins may also participate in regulating gene expression in addition to their established roles in cell division licensing.

## Materials and methods

### Bacterial strains and culture conditions

*V*. *cholerae* strains were routinely grown in LB medium and on LB agar supplemented when necessary with Carbenicillin (20 or 50 μg/mL), Kanamycin (50 μg/mL), Spectinomycin (200 μg/mL), Trimethoprim (10 μg/mL), and/or Chloramphenicol (2 μg/mL). For growth on a defined carbon source, strains were grown in M9 minimal medium containing the indicated carbon source.

### Transposon mutagenesis

Transposon mutant libraries were generated with a Carb^R^ mini-Tn10 transposon exactly as previously described [27]. Briefly, the transposon mutagenesis plasmid pDL1086 was first mated into the P_*chb*_-*lacZ* or P_*chb*_-*lacZ* Δ*cbp* reporter strain. Transposition was induced by plating cultures on Carb containing media at 42°C. To screen colonies, plates also contained 40 μg/mL X-gal and 5 mM IPTG. IPTG was added to competitively inhibit the basal activity of the P_*chb*_-*lacZ* reporter. This made it easier to distinguish colonies where the locus was uninduced (e.g. P_*chb*_-*lacZ*) from when the locus was induced (e.g. P_*chb*_-*lacZ* Δ*cbp*).

### Generation of mutant strains and constructs

The parent strain used in this study was E7946 [28]. Mutant constructs were generated using splicing-by-overlap extension PCR exactly as previously described [29]. Transformations and cotransformations were carried out exactly as previously described [30]. Mutant strains were confirmed by PCR and/or sequencing. The SlmA expression plasmid was generated by cloning the WT SlmA gene (VC0214) into the NdeI and BamHI sites of a His expression vector (pHis-tev). Untagged SlmA was also cloned into the EcoRI and BamHI sites of the IPTG-inducible expression vector pMMB67EH. Site directed mutants of SlmA were subsequently generated from these constructs using parallel single primer reactions exactly as previously described [31]. All plasmid inserts were confirmed by sequencing. Error prone PCR of SlmA was carried out exactly as previously described [32]. A detailed description of mutant strains and primers used in this study are outlined in **S1 Table** and **S2 Table**, respectively.

### Measuring GFP fluorescence

GFP was measured exactly as previously described [33]. Briefly, strains were grown at 30°C overnight, washed, and resuspended in instant ocean medium to a final OD_600_ of 1.0. Fluorescence was determined using a BioTek H1M plate reader with excitation set to 500 nm and emission at 540 nm.

### 5’ RACE

The 5’ end of transcripts were mapped using the SMARTer 5’/3’ RACE kit according to manufacturer’s instructions (Takara). The primers used are listed in **S2 Table**.

### Growth curves

Cells were grown in M9 minimal medium in the presence of 0.2% of the indicated carbon source at 37°C. Growth was kinetically monitored by measuring OD_600_ on a BioTek H1M plate reader.

### Quantitative reverse transcriptase PCR (qRT-PCR)

RNA was isolated from cells using an RNEasy minikit according to manufacturer’s instructions (Qiagen). RNA was reverse transcribed using AffinityScript QPCR cDNA Synthesis Kit (Agilent). Quantitative PCR was performed using iTaq Universal SYBR Green Supermix (Bio-Rad) with primers specific for the genes indicated (primers are listed in **S2 Table**) and the reaction was monitored on a Step-One qPCR system.

### Electrophoretic mobility shift assays (EMSAs)

EMSAs were performed essentially as previously described [29]. Briefly, probes were made by PCR. In the reaction we included Cy5-dCTP at a level that would result in incorporation of 1–2 Cy5 labeled nucleotides in the final probe. Binding reactions contained 20 mM Tris HCl pH 7.5, 100 mM KCl, 1 mM DTT, 5% glycerol, 0.1 mg/mL BSA, 0.1 mg/mL salmon sperm DNA, 2 nM Cy5 labeled DNA probe, and SlmA at the indicated concentrations in a 20 μL reaction volume. Reactions were incubated at room temperature for 30 minutes. Then, glycerol was added to a final concentration of 15% and 18 μL of each reaction was loaded onto a 5% polyacrylamide 0.5x TBE gel. The gel was run at 150V for 40 minutes in 0.5x TBE. Gels were imaged for Cy5 fluorescence on a Typhoon-9210 instrument or a BioRad ChemiDoc MP Imaging system.

### Western blot analysis

Cells were grown to mid-log in the presence of IPTG as an inducer where indicated. Cells were pelleted, resuspended and boiled in 1X SDS PAGE sample buffer and separated by polyacrylamide gel electrophoresis. Proteins were then transferred to PVDF and probed with rabbit polyconal α-FLAG (Sigma) or mouse monoclonal α-RpoA (Biolegend) primary antibodies. Blots were then incubated with α-rabbit or α-mouse secondary antibodies conjugated to IRdye 800CW as appropriate and imaged using an Odyssey classic LI-COR imaging system.

### Microscopy

Cells were grown to mid-log in LB medium and then mounted on 1% agarose pads. Cells were imaged using an Olympus IX83 phase contrast microscope.

## Supporting information

S1 Fig Levels of the long “uninduced” P_*chb*_ transcript do not change upon induction with (GlcNAc)_6_. Transcript levels were determined via qRT-PCR using primers specific for the long P_*chb*_ transcript. Data are shown as the mean ± SD and are from at least three independent biological replicates. **Fig 1D** shows the qRT-PCR data for the short “induced” transcript.
(PDF)

S2 Fig Mutant strains grow as expected on glucose, GlcNAc, and tryptone. Growth curves of the indicated strains in M9 minimal medium containing 0.5% (**A**) glucose, (**B**) tryptone, or (**C**) *N*-acetylglucosamine (GlcNAc) as a sole carbon source.
(PDF)

S3 Fig ChiS is required for growth on chitobiose. Growth curves of the indicated strains in M9 minimal medium containing 0.5% (**A**) chitiobiose, (**B**) *N*-acetylglucosamine, (**C**) glucose, or (**D**) tryptone as a sole carbon source.
(PDF)

S4 Fig Sequence alignment of SlmA protein from *V*. *cholerae* and *E*. *coli*. Residues highlighted in black are identical and those highlighted in gray are similar. Arrows indicate residues used in site-directed mutational analysis. T31 and E43 are involved in DNA-binding, F63 and R71 are involved in interaction with FtsZ, and R173 is involved in dimerization.
(PDF)

S5 Fig Truncations narrow the region of P_*chb*_ required for transcriptional activation. Promoters that were predicted computationally (Softberrry BPROM), are indicated as P1, P2, and P3, as well as a putative hairpin. All strains harbor the indicated region of P_*chb*_ fused to *gfp* to serve as a transcriptional reporter. Also, strains are either intact for *cbp* (+) or harbor a *cbp* deletion (Δ). Data are shown as the mean ± SD and are from at least three independent biological replicates.
(PDF)

S6 Fig An *Ec* SBS at P_*chb*_ does not enhance activation of P_*chb*_ by *Ec* SlmA. GFP fluorescence was measured in the indicated strains, all of which contain a P_*chb*_-*gfp* transcriptional reporter. “Ec SBS” indicates that the native SBS sequence in P_*chb*_ was swapped for the consensus SBS binding site for *Ec* SlmA. Data are from at least three independent biological replicates and shown as the mean ± SD. Please note that data from the first four bars is identical to that shown in **Fig 1E**, and are included here to allow for easy comparison to the additional samples included in this figure.
(PDF)

S7 Fig SlmA has a higher affinity for a synthetic SBS that represents the consensus sequence for SlmA binding in *E*. *coli* compared to the SBS present in P_*chb*_. (**A**) EMSAs performed where the indicated SBS sequence containing probes were incubated with (from left to right): 0 nM, 7.5 nM, 15 nM, 30 nM, 60 nM, 120 nM, and 240 nM of purified SlmA. Data are representative of at least two independent experiments. (**B**) Labeled *Vc* SBS from P_*chb*_ (0.2 nM) was incubated with increasing concentrations of unlabeled *Vc* SBS from P_*chb*_ or the consensus SBS from *E*. *coli*. Concentrations of unlabeled “cold competitor” probe from left to right are: 0 nM, 0.2 nM, 0.4 nM, 0.8 nM, 1.6 nM, 3.2 nM, 6.4 nM, 12.8 nM, 25.6 nM, 51.2 nM, and 200 nM.
(PDF)

S8 Fig Expression of SlmA mutant alleles is similar *in vivo*. (**A**) Fluorescence measurement of strains containing a P_*chb*_-*mcherry* reporter. Strains with *slmA*-3x FLAG were at the native *slmA* locus. Data indicates that the SlmA-3x FLAG construct is functional for P_*chb*_ activation. (**B**) SlmA site directed-mutants were engineered to contain a 3x FLAG tag at the native locus. Cell lysates of these strains were subjected to Western blot using a FLAG specific antibody and separately with an antibody to RpoA, which served as a loading control.
(PDF)

S9 Fig Mutations in SlmA that reduce DNA binding or FtsZ interaction diminish nucleoid occlusion activity. Strains containing chromosomally integrated constructs for overexpression of the indicated SlmA mutants via an IPTG-inducible P_*tac*_ promoter were grown in the presence or absence of 100 μM IPTG and imaged by phase contrast microscopy. Data are representative of at least two independent experiments.
(PDF)

S10 Fig Overexpression of SlmA and ChiS inhibits P_*chb*_ activation. (**A-D**) GFP fluorescence was assessed in strains that harbor a P_*chb*_-*gfp* reporter as well as a chromosomally integrated construct for overexpression (P_*tac*_-X) of ChiS and/or SlmA as indicated. All strains were grown with the indicated concentration of IPTG. P_*tac*_-*slmA* constructs contained a C-terminal triple FLAG tag, while P_*tac*_-*chiS* constructs were untagged because the FLAG tag diminished the activity of this protein. Data are shown as the mean ± SD and are from at least three independent biological replicates. (**E**) Representative western blots of SlmA-3x FLAG and ChiS-FLAG overexpression using the same chromosomally integrated constructs used in **A-D** for SlmA and a ChiS-FLAG construct. (**F**) Representative phase contrast images to show the morphology of cells ectopically expressing WT SlmA or SlmA F63A using the same mutant constructs and concentrations of IPTG used in **A-D**.
(PDF)

S11 Fig Overexpression of SlmA is not sufficient to activate expression of P_*chb*_. GFP fluorescence was assessed in strains that harbor a P_*chb*_-*gfp* reporter as well as the other mutations indicated. All strains were grown with 10 μM IPTG to overexpress the indicated SlmA mutant proteins. Consistent with a model where SlmA requires another coactivator, overexpression of these SlmA variants was not sufficient to induce expression of P_*chb*_. Data are shown as the mean ± SD and are from at least three independent biological replicates.
(PDF)

S12 Fig The placement of the SBS in P_*chb*_ is critical for transcriptional activation. All strains harbor a P_*chb*_-*gfp* reporter with the indicated mutation to move the SBS sequence as indicated. Each SBS mutation was tested in a background where *cbp* is intact (+) or deleted (Δ). Data are shown as the mean ± SD and are from at least three independent biological replicates.
(PDF)

S13 Fig Role of nonessential sigma factors in transcriptional activation of P_*chb*._ GFP fluorescence was assessed in strains that harbor a P_*chb*_-*gfp* reporter with the indicated sigma factor deleted. Data are shown as the mean ± SD and are from at least three independent biological replicates.
(PDF)

S1 Table Strains used in this study. (PDF)

S2 Table Primers used in this study. (PDF)
