# Supplementary figures and images for: The nucleoid occlusion protein SlmA is a direct transcriptional activator of chitobiose utilization in Vibrio cholerae
Source: PLoS Genet. 2017 Jul 6;13(7):e1006877. doi: 10.1371/journal.pgen.1006877 (PMC5519180; doi:10.1371/journal.pgen.1006877)

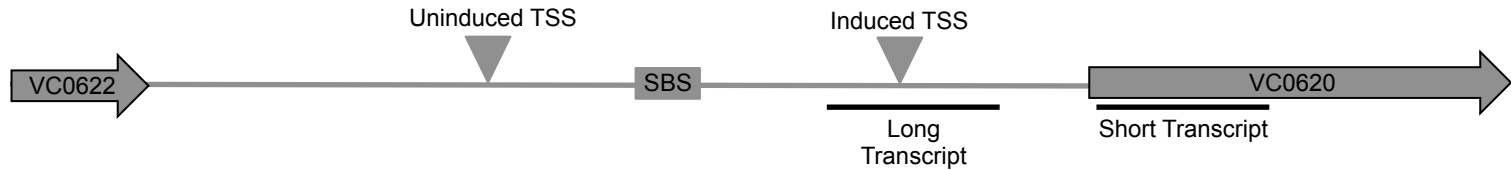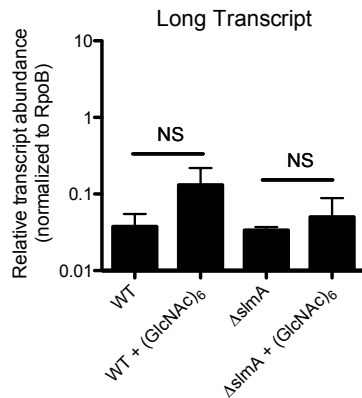

Supplement: S1 Fig — Transcript levels were determined via qRT-PCR using primers specific for the long Pchb transcript. Data are shown as the mean ± SD and are from at least three independent biological replicates. Fig 1D shows the qRT-PCR data for the short “induced” transcript. (PDF) [file pgen.1006877.s001.pdf]

**A**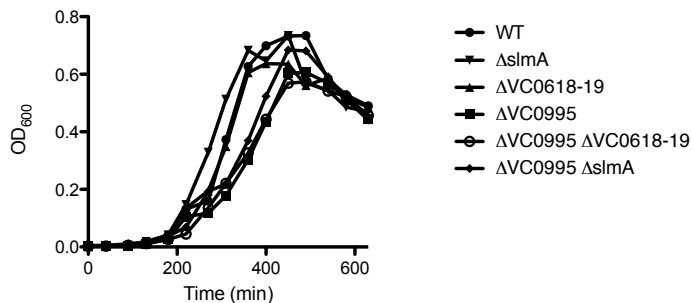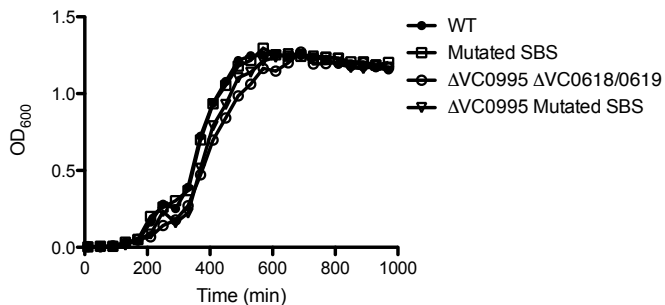**B**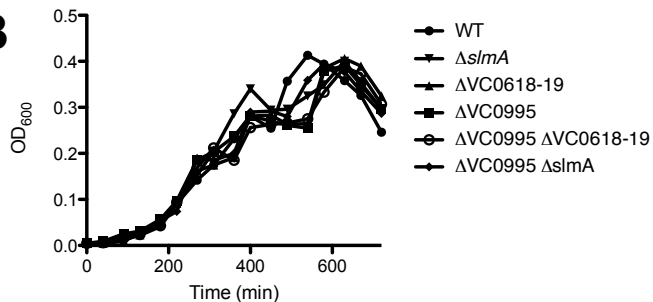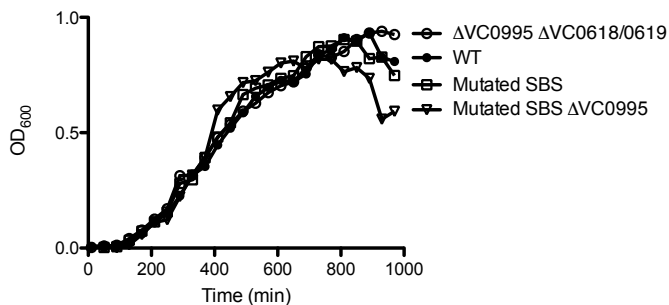**C**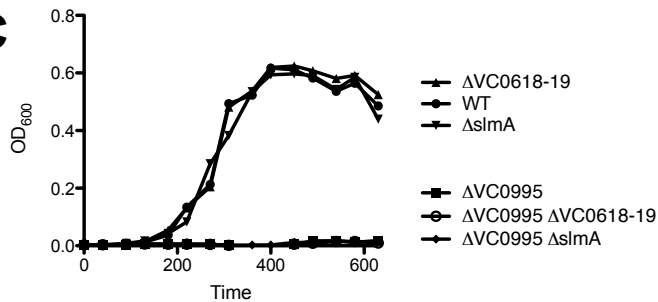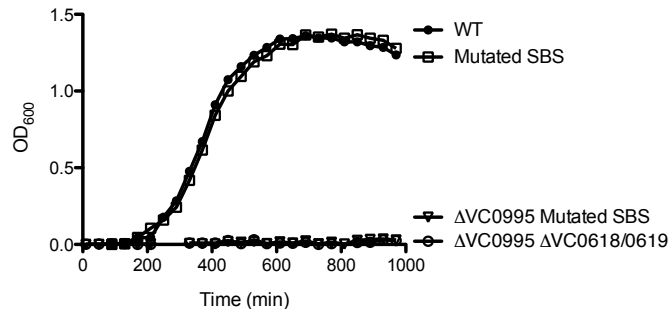

Supplement: S2 Fig — Growth curves of the indicated strains in M9 minimal medium containing 0.5% (A) glucose, (B) tryptone, or (C) N-acetylglucosamine (GlcNAc) as a sole carbon source. (PDF) [file pgen.1006877.s002.pdf]

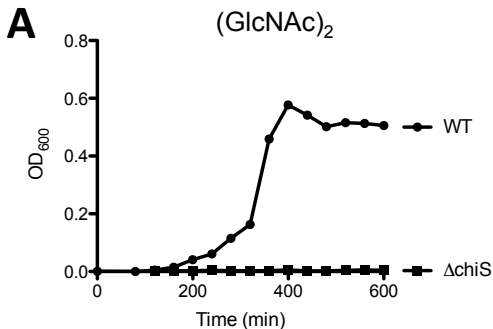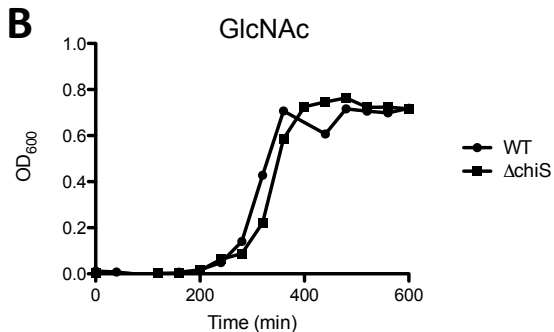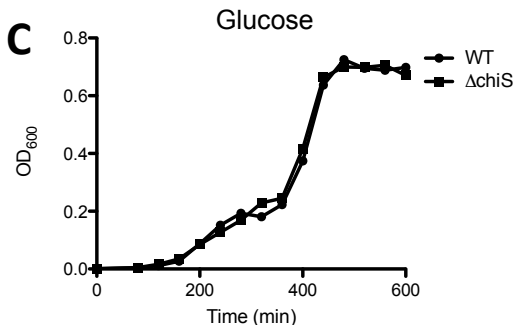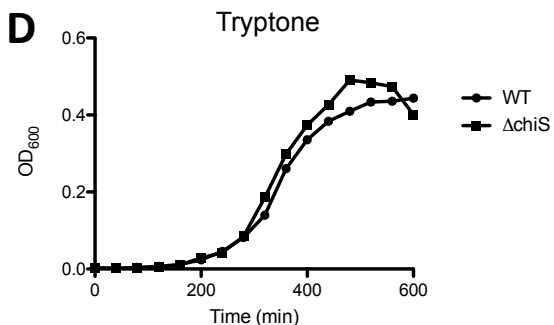

Supplement: S3 Fig — Growth curves of the indicated strains in M9 minimal medium containing 0.5% (A) chitiobiose, (B) N-acetylglucosamine, (C) glucose, or (D) tryptone as a sole carbon source. (PDF) [file pgen.1006877.s003.pdf]

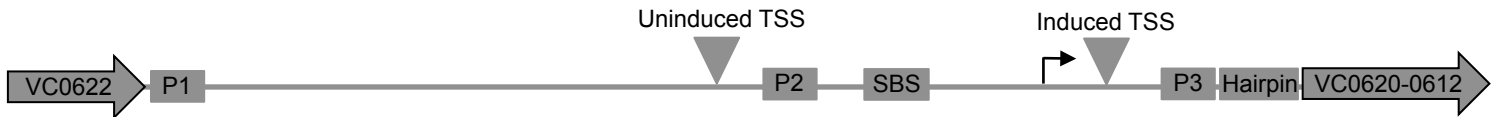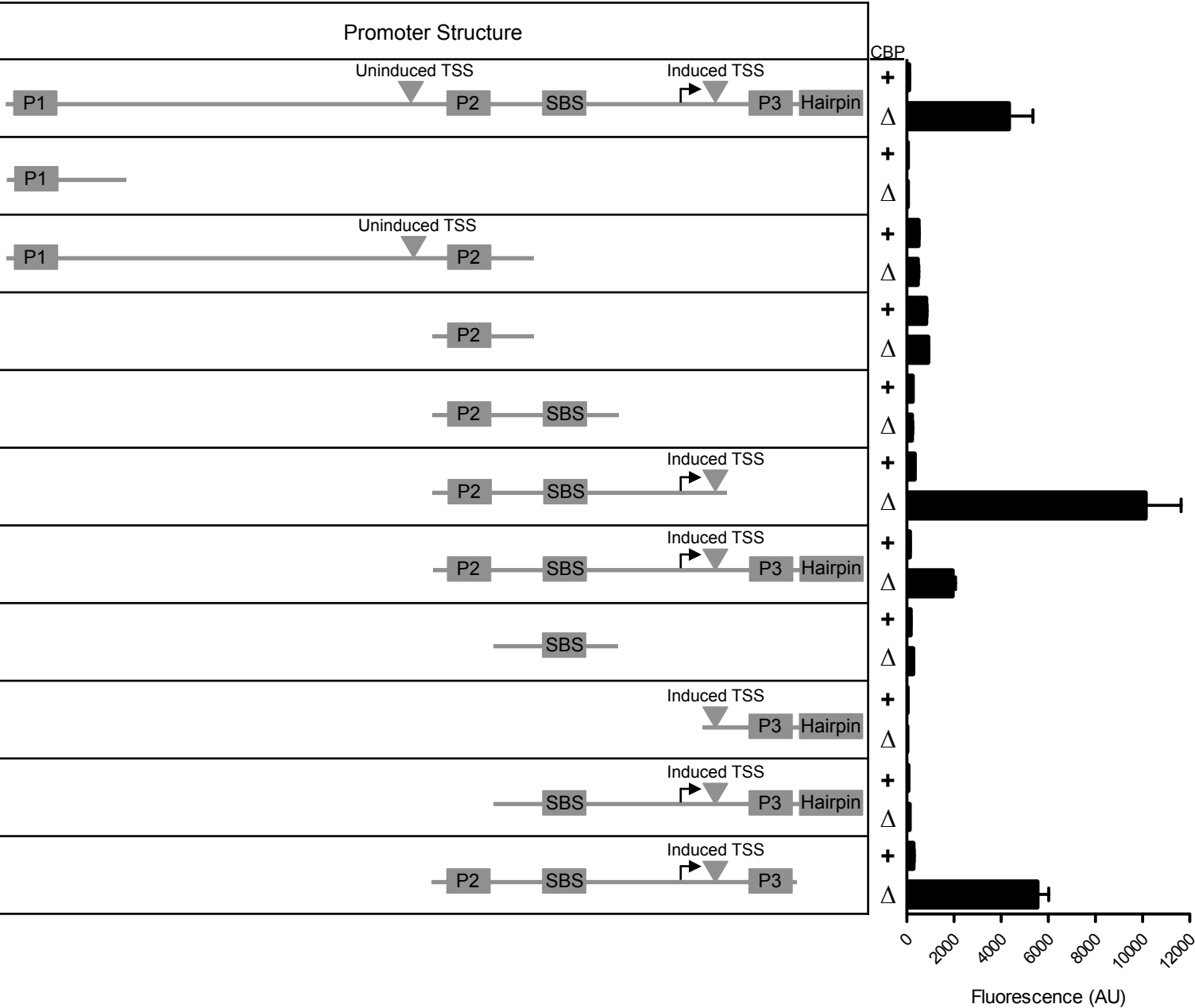

Supplement: S5 Fig — Promoters that were predicted computationally (Softberrry BPROM), are indicated as P1, P2, and P3, as well as a putative hairpin. All strains harbor the indicated region of Pchb fused to gfp to serve as a transcriptional reporter. Also, strains are either intact for cbp (+) or harbor a cbp deletion (Δ). Data are shown as the mean ± SD and are from at least three independent biological replicates. (PDF) [file pgen.1006877.s005.pdf]

$P_{chb}$ -GFP

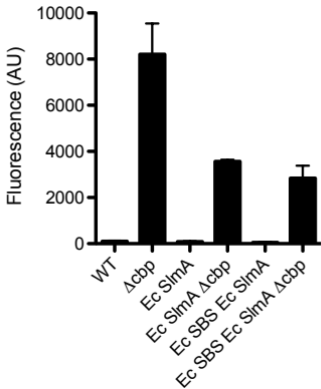

Supplement: S6 Fig — GFP fluorescence was measured in the indicated strains, all of which contain a Pchb-gfp transcriptional reporter. “Ec SBS” indicates that the native SBS sequence in Pchb was swapped for the consensus SBS binding site for Ec SlmA. Data are from at least three independent biological replicates and shown as the mean ± SD. Please note that data from the first four bars is identical to that shown in Fig 1E, and are included here to allow for easy comparison to the additional samples included in this figure. (PDF) [file pgen.1006877.s006.pdf]

**A**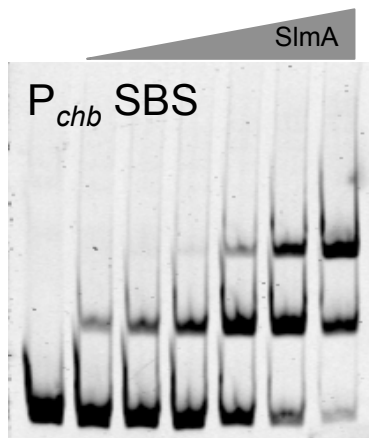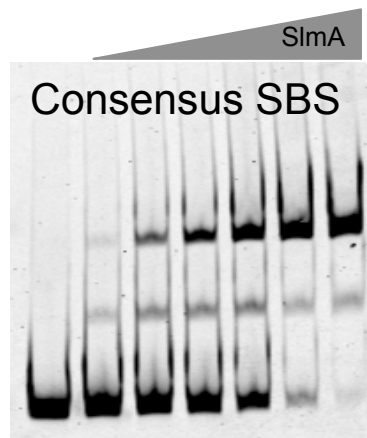**B**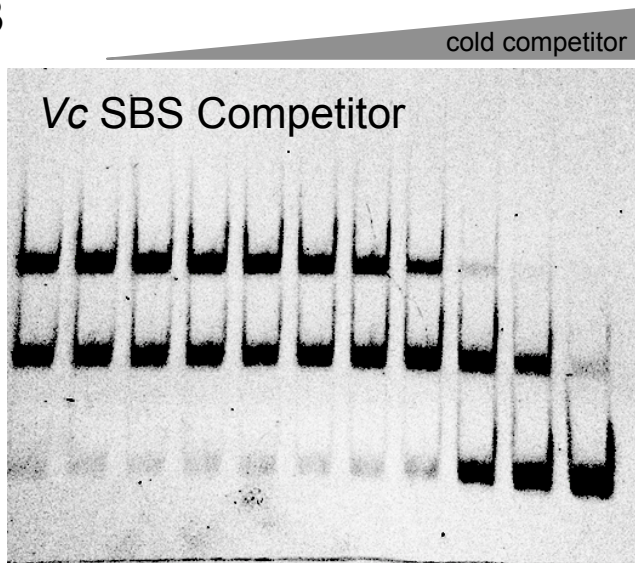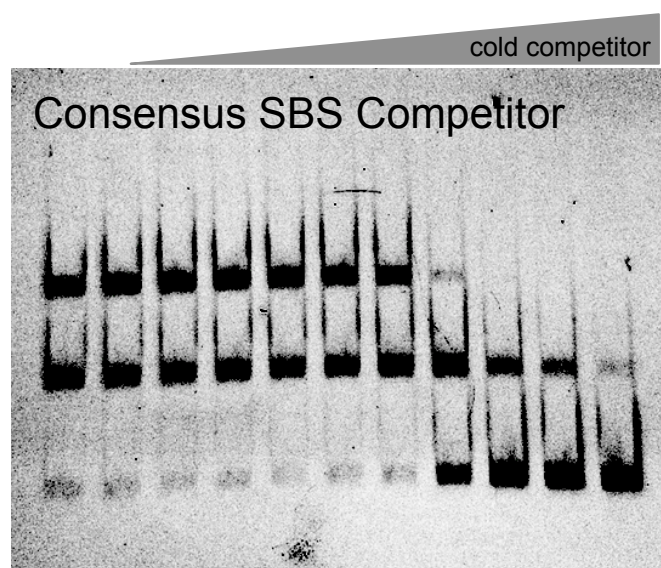

Supplement: S7 Fig — (A) EMSAs performed where the indicated SBS sequence containing probes were incubated with (from left to right): 0 nM, 7.5 nM, 15 nM, 30 nM, 60 nM, 120 nM, and 240 nM of purified SlmA. Data are representative of at least two independent experiments. (B) Labeled Vc SBS from Pchb (0.2 nM) was incubated with increasing concentrations of unlabeled Vc SBS from Pchb or the consensus SBS from E. coli. Concentrations of unlabeled “cold competitor” probe from left to right are: 0 nM, 0.2 nM, 0.4 nM, 0.8 nM, 1.6 nM, 3.2 nM, 6.4 nM, 12.8 nM, 25.6 nM, 51.2 nM, and 200 nM. (PDF) [file pgen.1006877.s007.pdf]

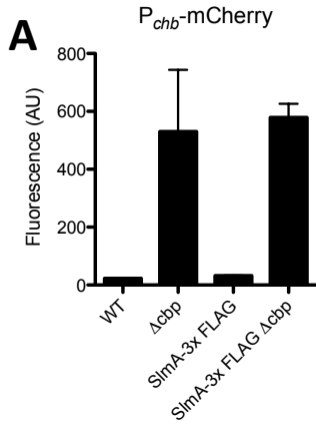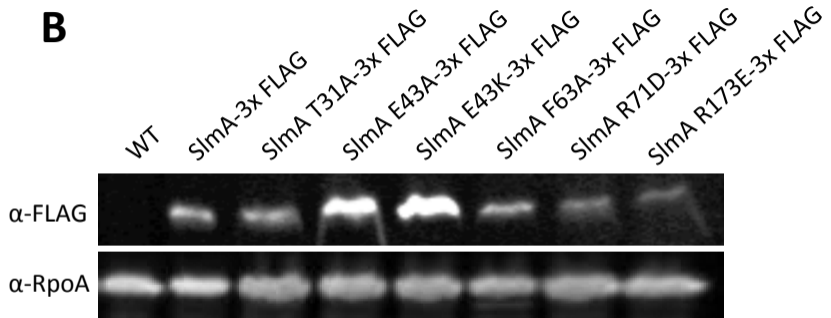

Supplement: S8 Fig — (A) Fluorescence measurement of strains containing a Pchb-mcherry reporter. Strains with slmA-3x FLAG were at the native slmA locus. Data indicates that the SlmA-3x FLAG construct is functional for Pchb activation. (B) SlmA site directed-mutants were engineered to contain a 3x FLAG tag at the native locus. Cell lysates of these strains were subjected to Western blot using a FLAG specific antibody and separately with an antibody to RpoA, which served as a loading control. (PDF) [file pgen.1006877.s008.pdf]

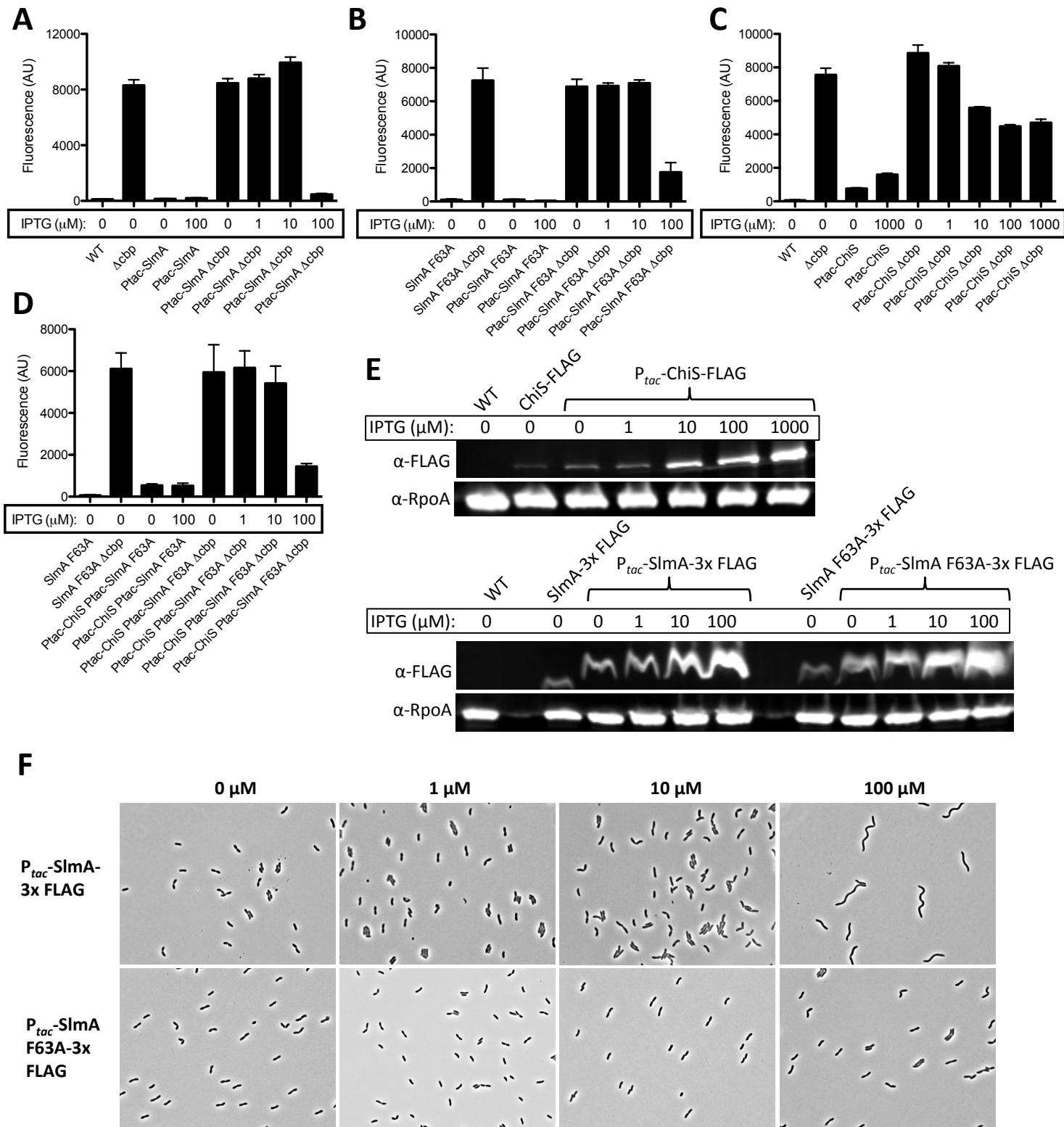

Supplement: S10 Fig — (A-D) GFP fluorescence was assessed in strains that harbor a Pchb-gfp reporter as well as a chromosomally integrated construct for overexpression (Ptac-X) of ChiS and/or SlmA as indicated. All strains were grown with the indicated concentration of IPTG. Ptac-slmA constructs contained a C-terminal triple FLAG tag, while Ptac-chiS constructs were untagged because the FLAG tag diminished the activity of this protein. Data are shown as the mean ± SD and are from at least three independent biological replicates. (E) Representative western blots of SlmA-3x FLAG and ChiS-FLAG overexpression using the same chromosomally integrated constructs used in A-D for SlmA and a ChiS-FLAG construct. (F) Representative phase contrast images to show the morphology of cells ectopically expressing WT SlmA or SlmA F63A using the same mutant constructs and concentrations of IPTG used in A-D. (PDF) [file pgen.1006877.s010.pdf]

$P_{chb}$ -GFP

$\Delta slmA$  background

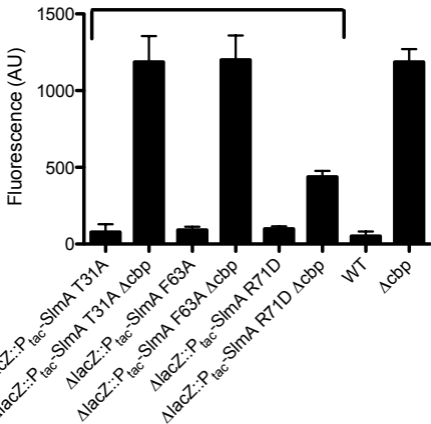

Supplement: S11 Fig — GFP fluorescence was assessed in strains that harbor a Pchb-gfp reporter as well as the other mutations indicated. All strains were grown with 10 μM IPTG to overexpress the indicated SlmA mutant proteins. Consistent with a model where SlmA requires another coactivator, overexpression of these SlmA variants was not sufficient to induce expression of Pchb. Data are shown as the mean ± SD and are from at least three independent biological replicates. (PDF) [file pgen.1006877.s011.pdf]

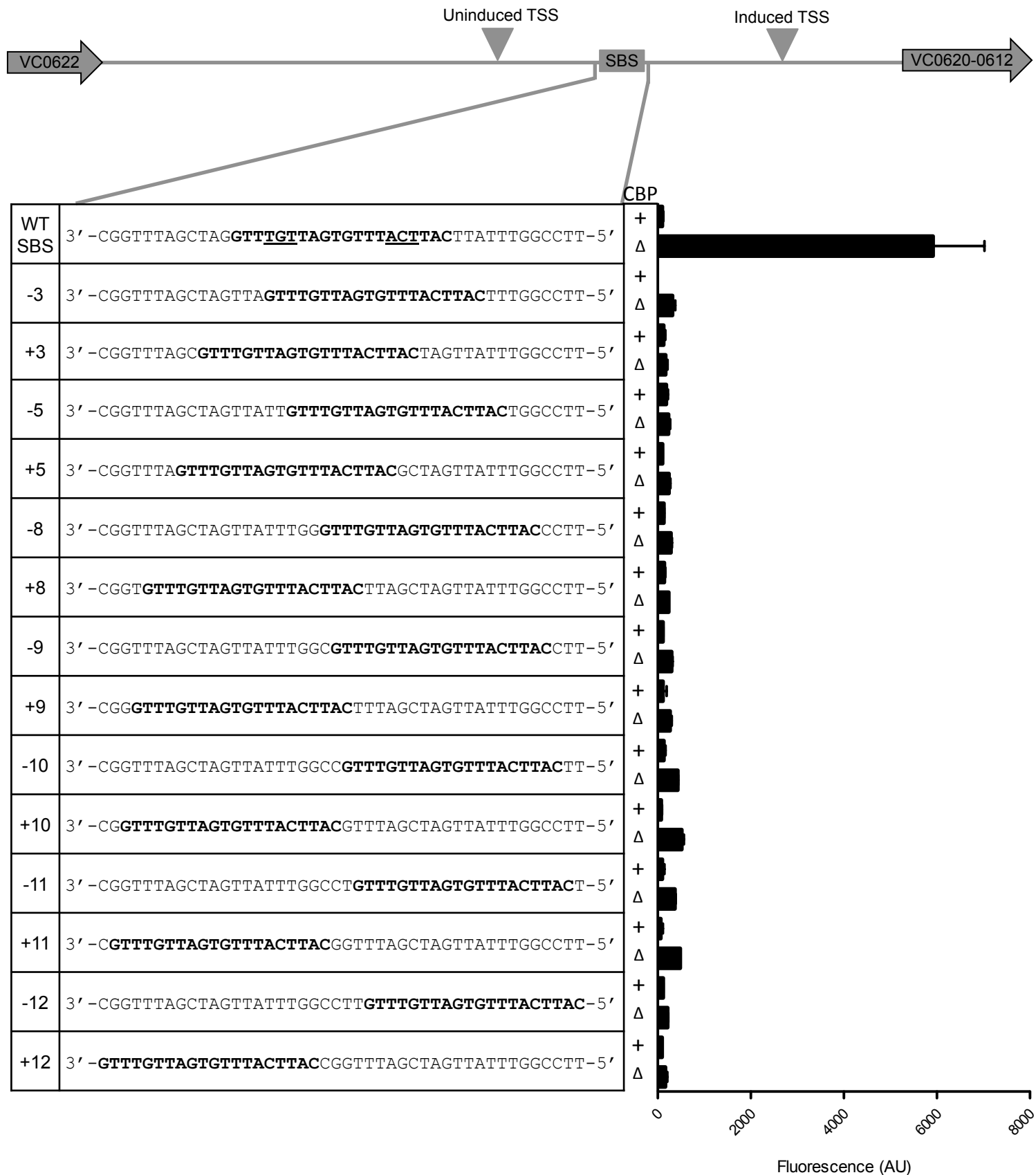

Supplement: S12 Fig — All strains harbor a Pchb-gfp reporter with the indicated mutation to move the SBS sequence as indicated. Each SBS mutation was tested in a background where cbp is intact (+) or deleted (Δ). Data are shown as the mean ± SD and are from at least three independent biological replicates. (PDF) [file pgen.1006877.s012.pdf]

$P_{chb}$ -GFP

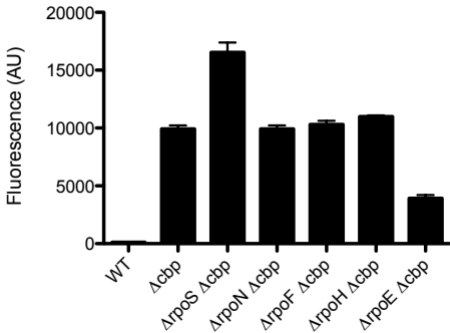

Supplement: S13 Fig — GFP fluorescence was assessed in strains that harbor a Pchb-gfp reporter with the indicated sigma factor deleted. Data are shown as the mean ± SD and are from at least three independent biological replicates. (PDF) [file pgen.1006877.s013.pdf]
