# Supplementary material for: The nucleoid occlusion protein SlmA is a direct transcriptional activator of chitobiose utilization in Vibrio cholerae
Source: PLoS Genet. 2017 Jul 6;13(7):e1006877. doi: 10.1371/journal.pgen.1006877 (PMC5519180; doi:10.1371/journal.pgen.1006877)
Supplement: S4 Fig — Residues highlighted in black are identical and those highlighted in gray are similar. Arrows indicate residues used in site-directed mutational analysis. T31 and E43 are involved in DNA-binding, F63 and R71 are involved in interaction with FtsZ, and R173 is involved in dimerization. (PDF) [file pgen.1006877.s004.pdf]

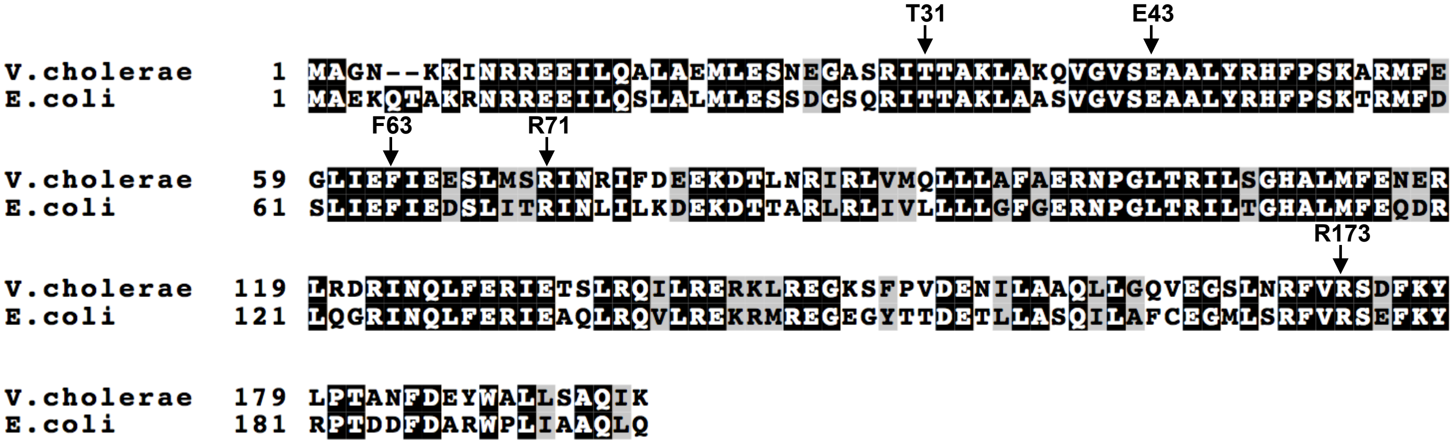
T31
E43
V.cholerae 1 MAGN--KKINRREEILQALAEMLESNEGASRITTAKLAKQVGVSEAALYRHFPSKARMFE
E.coli 1 MAEKQTAKRNRREEILQSLALMLESSDGSQRITTAKLAASVGVSEAALYRHFPSKTRMFD
F63
R71
V.cholerae 59 GLIEFIEESLMSRINRIFDEEKDTLNRIRLVMQLLLAFAERNPGLTRILSGHALMFENER
E.coli 61 SLIEFIEDSLITRINLILKDEKDTTARLRLIVLLLLGFGERNPGLTRILTGHALMFEQDR
R173
V.cholerae 119 LRDRINQLFERIETSLRQILRERKLREGKSFPVDENILAAQLLGQVEGSLNRFVRSDFKY
E.coli 121 LQGRINQLFERIEAQLRQVLREKRMREGEGYTTDETLLASQILAFCEGMLSRFVRSEFKY
V.cholerae 179 LPTANFDEYWALLSAQIK
E.coli 181 RPTDDFDARWPLIAAQLQ
