# Supplementary material for: The nucleoid occlusion protein SlmA is a direct transcriptional activator of chitobiose utilization in Vibrio cholerae
Source: PLoS Genet. 2017 Jul 6;13(7):e1006877. doi: 10.1371/journal.pgen.1006877 (PMC5519180; doi:10.1371/journal.pgen.1006877)
Supplement: S9 Fig — Strains containing chromosomally integrated constructs for overexpression of the indicated SlmA mutants via an IPTG-inducible Ptac promoter were grown in the presence or absence of 100 μM IPTG and imaged by phase contrast microscopy. Data are representative of at least two independent experiments. (PDF) [file pgen.1006877.s009.pdf]

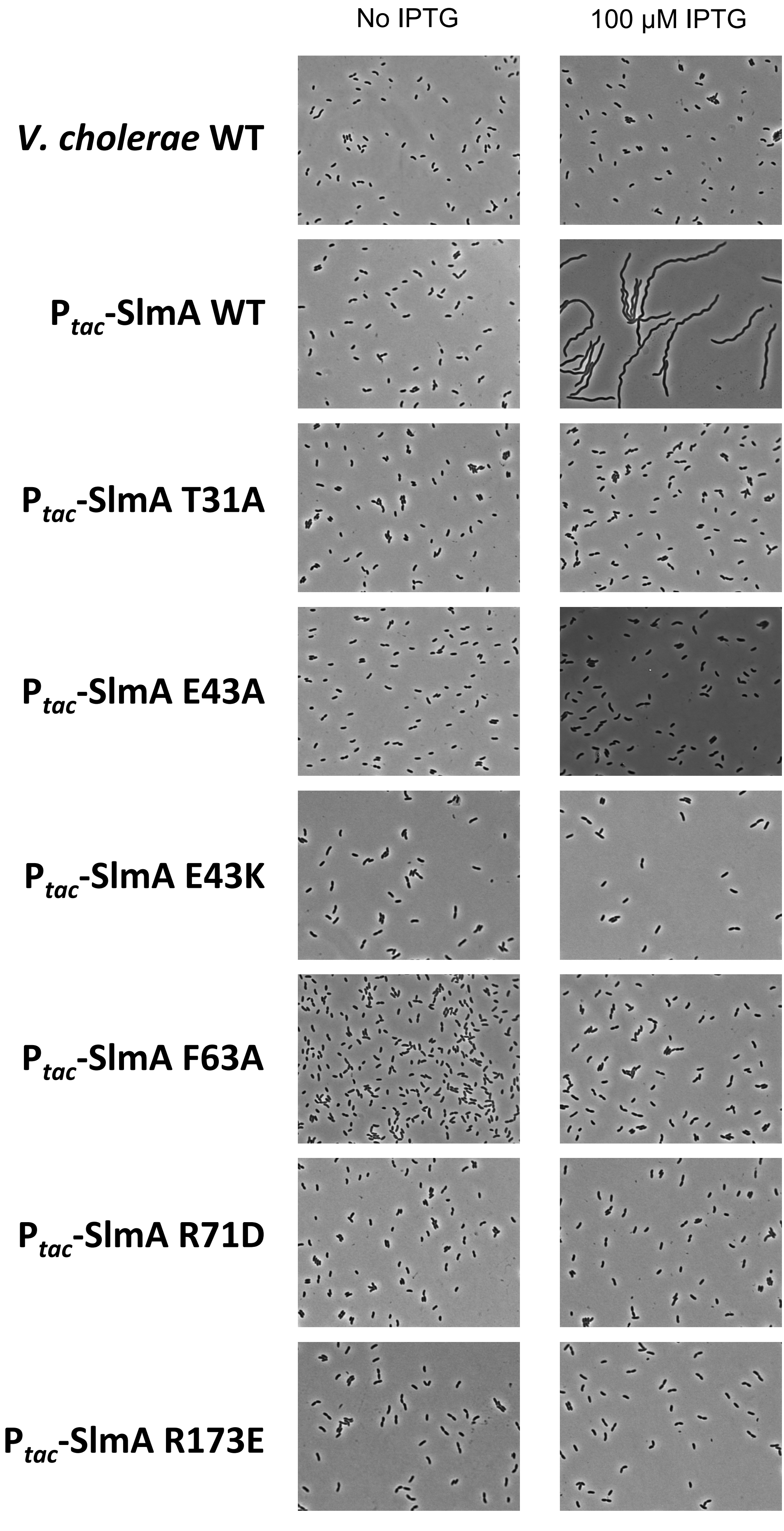
No IPTG
100 μM IPTG
V. cholerae WT
Ptac-SlmA WT
Ptac-SlmA T31A
Ptac-SlmA E43A
Ptac-SlmA E43K
Ptac-SlmA F63A
Ptac-SlmA R71D
Ptac-SlmA R173E
