# Supplementary material for: The nucleoid occlusion protein SlmA is a direct transcriptional activator of chitobiose utilization in Vibrio cholerae
Source: PLoS Genet. 2017 Jul 6;13(7):e1006877. doi: 10.1371/journal.pgen.1006877 (PMC5519180; doi:10.1371/journal.pgen.1006877)
Supplement: S1 Table — (PDF) [file pgen.1006877.s014.pdf]

Table S1 – *Strains used in this study*

Strain name in manuscript	Genotype and antibiotic resistances	Description	Reference / (strain#)
WT	E7946 Sm^R	Wildtype *V. cholerae* O1 El Tor strain used throughout this study	(25) (SAD030)
**Strains Used for Transposon Library**			
Parent strain used for repressor transposon library	P_{chb}-*lacZ*, Kan^R; pDL1086 Amp^R Cm^R	CAK 062 parent harboring transposon vector pDL1086	This Study (CAK 065 / SAD 1397)
Parent strain used for activator transposon library	P_{chb}-*lacZ*, Kan^R; ΔCBP::$Spec^R$; pDL1086 Amp^R Cm^R	CAK 063 parent harboring transposon vector pDL1086	This Study (CAK 066 / SAD 1398)
**Strains Used for Growth Curves**			
ΔSlmA	Δ*slmA*::Kan^R	Deletion of *slmA* replaced with Kan^R	This Study (CAK 021 / SAD 1392)
ΔVC0995	ΔVC1807::$Spec^R$; ΔVC0995	A deletion of VC0995 GlcNAc monosaccharide transporter and a deletion of VC1807 replaced with $Spec^R$ cassette	This Study (SAD 265)
ΔVC0995 ΔVC0618-19	ΔVC1807::$Spec^R$; ΔVC0995; ΔVC0618-19::Kan^R	SAD 265 parent with a deletion of VC0618 and VC0619 replaced with Kan^R	This Study (CAK 072 / SAD 1399)
ΔVC0995 Δ*slmA*	ΔVC1807::$Spec^R$; ΔVC0995; Δ*slmA*::Tm^R	SAD 265 parent with a deletion of *slmA* replaced with Tm^R	This Study (CAK 073 / SAD 1400)
ΔVC0618-19	ΔVC0618-19::Kan^R	Deletion of VC0618 and VC0619 replaced with KanR	This Study (SAD 382)
Scrambled SBS	P_{chb} Scrambled SBS; ΔVC1807::Kan^R	SBS at native P_{chb} promoter was scrambled and VC1807 was replaced with Kan^R	This Study (CAK 172 / SAD 1439)
Scrambled SBS ΔVC0995	P_{chb} Scrambled SBS; ΔVC1807::Kan^R; ΔVC0995	SAD 265 parent; SBS at native promoter was scrambled and Kan^R cassette swapped for $Spec^R$ cassette at VC1807	This Study (CAK 173 / SAD 1440)
Δ*chiS*	Δ*chiS*::$Spec^R$	A deletion of *chiS* replaced with $Spec^R$	This study (SAD 117)
**Strains Used for LacZ Activity**			
Parent LacZ reporter	P_{chb}-*lacZ*, Kan^R	P_{chb}-*lacZ* transcriptional reporter	This Study (CAK 062 / SAD 1395)
ΔCBP LacZ reporter	P_{chb}-*lacZ*, Kan^R; ΔCBP::$Spec^R$	P_{chb}-*lacZ* transcriptional reporter with a deletion of CBP replaced with $Spec^R$ cassette	This Study (CAK 063 / SAD 1396)
ΔCBP Δ*slmA* LacZ reporter	P_{chb}-*lacZ*, Kan^R; ΔCBP::$Spec^R$; Δ*slmA*::Tm^R	CAK 062 parent with a deletion of *slmA* replaced with Tm^R and a deletion of CBP replaced with $Spec^R$ cassette	This Study (CAK 075 / SAD 1401)
ΔChiS ΔCBP LacZ reporter	P_{chb}-*lacZ*, Kan^R; ΔChiS::TmR; ΔCBP::$Spec^R$	CAK 062 parent with a deletion of ChiS replaced with Tm^R and a deletion of CBP replaced with $Spec^R$	This Study (CAK 209 / SAD 1460)
**Strains Used for P_{chb}-GFP Assays**			

WT GFP reporter, WT SBS GFP reporter	Δ*lacZ*::P_{chb}-GFP Kan^R	A deletion of *lacZ* replaced with P_{chb}-GFP transcriptional fusion linked to Kan^R	This Study (CAK 007 / SAD 590)
ΔCBP GFP reporter, WT SBS ΔCBP GFP reporter	Δ*lacZ*::P_{chb}-GFP Kan^R; ΔCBP::$Spec^R$	CAK 007 parent with a deletion of CBP replaced with $Spec^R$ cassette	This Study (CAK 009 / SAD 1390)
ΔCBP ΔChiS GFP reporter	Δ*lacZ*::P_{chb}-GFP Kan^R; ΔCBP::$Spec^R$; ΔChiS::Tm^R	CAK 007 parent with a deletion of CBP replaced with $Spec^R$ cassette and a deletion of ChiS replaced with Tm^R cassette	This Study (CAK 010 / SAD 1391)
ΔSlmA GFP reporter	Δ*lacZ*::P_{chb}-GFP, Kan^R; Δ*slmA*::Tm^R	CAK 007 parent with a deletion of *slmA* replaced with Tm^R cassette	This Study (CAK 024 / SAD 1393)
ΔSlmA ΔCBP GFP Reporter	Δ*lacZ*::P_{chb}-GFP, Kan^R; Δ*slmA*::Tm^R; ΔCBP::$Spec^R$	CAK 007 parent with a deletion of *slmA* replaced with Tm^R cassette and a deletion of CBP replaced with $Spec^R$ cassette	This Study (CAK 025 / 1394)
Scrambled SBS	Δ*lacZ*::P_{chb}-GFP with scrambled SBS	A deletion of *lacZ* replaced with P_{chb}-GFP transcriptional fusion linked to Kan^R; SBS in promoter element was “scrambled”	This Study (CAK 088 / SAD 1402)
pMMB SlmA ΔCBP	Δ*lacZ*::P_{chb}-GFP, Kan^R; Δ*slmA*::Tm^R; ΔCBP::$Spec^R$; pMMB67EH SlmA	CAK 025 parent harboring pMMB67EH with *slmA* insert	This Study (CAK 299 / SAD 1480)
pMMB ΔCBP	Δ*lacZ*::P_{chb}-GFP, Kan^R; Δ*slmA*::Tm^R; ΔCBP::$Spec^R$; pMMB67EH empty vector	CAK 025 parent harboring pMMB67EH with no insert	This Study (CAK 300 / SAD 1481)
Scrambled SBS ΔCBP	Δ*lacZ*::P_{chb}-GFP with scrambled SBS; ΔCBP::$Spec^R$	A deletion of *lacZ* replaced with P_{chb}-GFP transcriptional fusion linked to Kan^R; SBS in promoter element was “scrambled”; a deletion of CBP replaced with $Spec^R$	This Study (CAK 089 / SAD 1403)
WT SlmA GFP reporter	Δ*lacZ*::P_{chb}-GFP, Kan^R; ΔVC1807::$Spec^R$	CAK 024 parent with WT SlmA knocked back in at native locus; has the same phenotype as CAK 007	This Study (CAK 093 / SAD 1404)
WT SlmA ΔCBP GFP reporter	Δ*lacZ*::P_{chb}-GFP, Kan^R; ΔCBP::$Spec^R$	CAK 024 parent with WT SlmA knocked back in at native locus and a deletion of CBP replaced with $Spec^R$; has the same phenotype as CAK 009	This Study (CAK 094 / SAD 1405)
SlmA T31A GFP reporter	Δ*lacZ*::P_{chb}-GFP, Kan^R; ΔVC1807::$Spec^R$; SlmA T31A	CAK 024 parent with SlmA T31A knocked back in at native locus	This Study (CAK 095 / SAD 1406)
SlmA T31A ΔCBP GFP reporter	Δ*lacZ*::P_{chb}-GFP, Kan^R; ΔCBP::$Spec^R$; SlmA T31A	CAK 024 parent with SlmA T31A knocked back in at native locus and a deletion of CBP replaced with $Spec^R$	This Study (CAK 096 / SAD 1407)
SlmA E43A GFP reporter	Δ*lacZ*::P_{chb}-GFP, Kan^R; ΔVC1807::$Spec^R$; SlmA E43A	CAK 024 parent with SlmA E43A knocked back in at native locus	This Study (CAK 143 / SAD 1430)

SlmA E43A ΔCBP GFP reporter	Δ*lacZ*::P_{chb}-GFP, Kan^R; ΔCBP::$Spec^R$; SlmA E43A	CAK 024 parent with SlmA E43A knocked back in at native locus and a deletion of CBP replaced with $Spec^R$	This Study (CAK 144 / SAD 1431)
SlmA F63A GFP reporter	Δ*lacZ*::P_{chb}-GFP, Kan^R; ΔVC1807::$Spec^R$; SlmA F63A	CAK 024 parent with SlmA F63A knocked back in at native locus	This Study (CAK 097 / SAD 1408)
SlmA F63A ΔCBP GFP reporter	Δ*lacZ*::P_{chb}-GFP, Kan^R; ΔCBP::$Spec^R$; SlmA F63A	CAK 024 parent with SlmA F63A knocked back in at native locus and a deletion of CBP replaced with $Spec^R$	This Study (CAK 098 / SAD 1409)
SlmA R71D GFP reporter	Δ*lacZ*::P_{chb}-GFP, Kan^R; ΔVC1807::$Spec^R$; SlmA R71D	CAK 024 parent with SlmA R71D knocked back in at native locus	This Study (CAK 099 / SAD 1410)
SlmA R71D ΔCBP GFP reporter	Δ*lacZ*::P_{chb}-GFP, Kan^R; ΔCBP::$Spec^R$; SlmA R71D	CAK 024 parent with SlmA R71D knocked back in at native locus and a deletion of CBP replaced with $Spec^R$	This Study (CAK 108 / SAD 1413)
SlmA R173E GFP reporter	Δ*lacZ*::P_{chb}-GFP, Kan^R; ΔVC1807::$Spec^R$; SlmA R173E	CAK 024 parent with SlmA R173E knocked back in at native locus	This Study (CAK 193 / SAD 1452)
SlmA R173E ΔCBP GFP reporter	Δ*lacZ*::P_{chb}-GFP, Kan^R; ΔCBP::$Spec^R$; SlmA R173E	CAK 024 parent with SlmA R173E knocked back in at native locus and a deletion of CBP replaced with $Spec^R$	This Study (CAK 194 / SAD 1453)
Ec SlmA GFP reporter	Δ*lacZ*::P_{chb}-GFP, Kan^R; ΔVC1807::$Spec^R$; Ec SlmA	CAK 024 parent with SlmA from *E. coli* knocked back in at native locus	This Study (CAK 100 / SAD 1411)
Ec SlmA ΔCBP GFP reporter	Δ*lacZ*::P_{chb}-GFP, Kan^R; ΔCBP::$Spec^R$; Ec SlmA	CAK 024 parent with SlmA from *E. coli* knocked back in at native locus and a deletion of CBP replaced with $Spec^R$	This Study (CAK 101 / SAD 1412)
Ec SBS GFP reporter	Δ*lacZ*::P_{chb}-GFP with Ec SBS	A deletion of *lacZ* replaced with P_{chb}-GFP transcriptional fusion linked to Kan^R; SBS in promoter element was replaced with *E. coli* consensus SBS	This Study (CAK 168 / SAD 1438)
Ec SBS GFP reporter ΔCBP	Δ*lacZ*::P_{chb}-GFP with Ec SBS; ΔCBP::$Spec^R$	CAK 168 parent with a deletion of CBP replaced with $Spec^R$	This Study (CAK 197 / SAD 1455)
P1	Δ*lacZ*::P1 of P_{chb}-GFP, Kan^R	P_{chb} truncation fused to GFP linked to Kan^R	This Study (SAD 903)
P1 ΔCBP	Δ*lacZ*::P1 of P_{chb}-GFP, Kan^R; ΔCBP::$Spec^R$	P_{chb} truncation fused to GFP linked to Kan^R and a deletion of CBP replaced with $Spec^R$	This Study (SAD 904)
P1+P2	Δ*lacZ*::P1+P2 of P_{chb}-GFP, Kan^R	P_{chb} truncation fused to GFP linked to Kan^R	This Study (SAD 912)
P1+P2 ΔCBP	Δ*lacZ*::P1+P2 of P_{chb}-GFP, Kan^R; ΔCBP::$Spec^R$	P_{chb} truncation fused to GFP linked to Kan^R and a deletion of CBP replaced with $Spec^R$	This Study (SAD 913)
P2 only	Δ*lacZ*::P2 only of P_{chb}-GFP, $Spec^R$	P_{chb} truncation fused to GFP linked to $Spec^R$	This Study (CAK 111 / SAD 1415)

P2 only ΔCBP	Δ*lacZ*::P2 only of P_{chb}-GFP; ΔCBP::Tm^R	P_{chb} truncation fused to GFP linked to $Spec^R$ and a deletion of CBP replaced with Tm^R	This Study (CAK 112 / SAD 1416)
P2+SBS	Δ*lacZ*::P2+SBS of P_{chb}-GFP, $Spec^R$	P_{chb} truncation fused to GFP linked to $Spec^R$	This Study (CAK 113 / SAD 1417)
P2+SBS ΔCBP	Δ*lacZ*::P2+SBS of P_{chb}-GFP, $Spec^R$; ΔCBP::Tm^R	P_{chb} truncation fused to GFP linked to $Spec^R$ and a deletion of CBP replaced with Tm^R	This Study (CAK 114 / SAD 1418)
P2+SBS more space	Δ*lacZ*::P2+SBS more space of P_{chb}-GFP, $Spec^R$	P_{chb} truncation fused to GFP linked to $Spec^R$	This Study (CAK 116 / SAD 1419)
P2+SBS more space ΔCBP	Δ*lacZ*::P2+SBS more space of P_{chb}-GFP, $Spec^R$; ΔCBP::Tm^R	P_{chb} truncation fused to GFP linked to $Spec^R$ and a deletion of CBP replaced with Tm^R	This Study (CAK 117 / SAD 1420)
P2+P3	Δ*lacZ*::P2+P3 of P_{chb}-GFP, Kan^R	P_{chb} truncation fused to GFP linked to Kan^R	This Study (SAD 907)
P2+P3 ΔCBP	Δ*lacZ*::P2+P3 of P_{chb}-GFP, Kan^R; ΔCBP::$Spec^R$	P_{chb} truncation fused to GFP linked to Kan^R and a deletion of CBP replaced with $Spec^R$	This Study (SAD 908)
P2+P3 internal region	Δ*lacZ*::P2+P3 internal region of P_{chb}-GFP, $Spec^R$	P_{chb} truncation fused to GFP linked to $Spec^R$	This Study (CAK 129 / SAD 1425)
P2+P3 internal region ΔCBP	Δ*lacZ*::P2+P3 internal region of P_{chb}-GFP, $Spec^R$; ΔCBP::Tm^R	P_{chb} truncation fused to GFP linked to $Spec^R$ and a deletion of CBP replaced with Tm^R	This Study (CAK 130 / SAD 1426)
P3	Δ*lacZ*::P3 of P_{chb}-GFP, Kan^R	P_{chb} truncation fused to GFP linked to Kan^R	This Study (SAD 905)
P3 ΔCBP	Δ*lacZ*::P3 of P_{chb}-GFP, Kan^R; ΔCBP::$Spec^R$	P_{chb} truncation fused to GFP linked to Kan^R and a deletion of CBP replaced with $Spec^R$	This Study (SAD 906)
P3 only more space	Δ*lacZ*::P3 only more space of P_{chb}-GFP, $Spec^R$	P_{chb} truncation fused to GFP linked to $Spec^R$	This Study (CAK 119 / SAD 1421)
P3 only more space ΔCBP	Δ*lacZ*::P3 only more space of P_{chb}-GFP, $Spec^R$; ΔCBP::Tm^R	P_{chb} truncation fused to GFP linked to $Spec^R$ and a deletion of CBP replaced with Tm^R	This Study (CAK 120 / SAD 1422)
ΔHairpin	Δ*lacZ*::Δhairpin of P_{chb}-GFP, $Spec^R$	P_{chb} truncation fused to GFP linked to $Spec^R$	This Study (CAK 121 / SAD 1423)
ΔHairpin ΔCBP	Δ*lacZ*::Δhairpin of P_{chb}-GFP, $Spec^R$; ΔCBP::Tm^R	P_{chb} truncation fused to GFP linked to $Spec^R$ and a deletion of CBP replaced with Tm^R	This Study (CAK 122 / SAD 1424)
-3 SBS	Δ*lacZ*::P_{chb}-GFP, Kan^R; -3 SBS	A deletion of *lacZ* replaced with P_{chb}-GFP transcriptional fusion linked to Kan^R; SBS in promoter element was moved 3 bp downstream	This Study (CAK 179 / SAD 1441)
-3 SBS ΔCBP	Δ*lacZ*::P_{chb}-GFP, Kan^R; -3 SBS; ΔCBP::$Spec^R$	A deletion of *lacZ* replaced with P_{chb}-GFP transcriptional fusion linked to Kan^R; SBS in promoter element was moved 3 bp downstream; a deletion of CBP was replaced with $Spec^R$	This Study (CAK 180 / SAD 1442)

+3 SBS	Δ*lacZ*::P_{chb}-GFP, Kan^R; +3 SBS	A deletion of *lacZ* replaced with P_{chb}-GFP transcriptional fusion linked to Kan^R; SBS in promoter element was moved 3 bp upstream	This Study (CAK 183 / SAD 1445)
+3 SBS ΔCBP	Δ*lacZ*::P_{chb}-GFP, Kan^R; +3 SBS; ΔCBP::$Spec^R$	A deletion of *lacZ* replaced with P_{chb}-GFP transcriptional fusion linked to Kan^R; SBS in promoter element was moved 3 bp upstream; a deletion of CBP was replaced with $Spec^R$	This Study (CAK 184 / SAD 1446)
-5 SBS	Δ*lacZ*::P_{chb}-GFP, Kan^R; -5 SBS	A deletion of *lacZ* replaced with P_{chb}-GFP transcriptional fusion linked to Kan^R; SBS in promoter element was moved 5 bp downstream	This Study (CAK 181 / SAD 1443)
-5 SBS ΔCBP	Δ*lacZ*::P_{chb}-GFP, Kan^R; -5 SBS; ΔCBP::$Spec^R$	A deletion of *lacZ* replaced with P_{chb}-GFP transcriptional fusion linked to Kan^R; SBS in promoter element was moved 5 bp downstream; a deletion of CBP was replaced with $Spec^R$	This Study (CAK 182 / SAD 1444)
+5 SBS	Δ*lacZ*::P_{chb}-GFP, Kan^R; +5 SBS	A deletion of *lacZ* replaced with P_{chb}-GFP transcriptional fusion linked to Kan^R; SBS in promoter element was moved 5 bp upstream	This Study (CAK 185 / SAD 1447)
+5 SBS ΔCBP	Δ*lacZ*::P_{chb}-GFP, Kan^R; +5 SBS; ΔCBP::$Spec^R$	A deletion of *lacZ* replaced with P_{chb}-GFP transcriptional fusion linked to Kan^R; SBS in promoter element was moved 5 bp upstream; a deletion of CBP was replaced with $Spec^R$	This Study (CAK 186 / SAD 1448)
-8 SBS	Δ*lacZ*::P_{chb}-GFP, Kan^R; -8 SBS	A deletion of *lacZ* replaced with P_{chb}-GFP transcriptional fusion linked to Kan^R; SBS in promoter element was moved 8 bp downstream	This Study (CAK 217 / SAD 1463)
-8 SBS ΔCBP	Δ*lacZ*::P_{chb}-GFP, Kan^R; -8 SBS; ΔCBP::$Spec^R$	A deletion of *lacZ* replaced with P_{chb}-GFP transcriptional fusion linked to Kan^R; SBS in promoter element was moved 8 bp downstream; a deletion of CBP replaced with $Spec^R$	This Study (CAK 225 / SAD 1471)
+8 SBS	Δ*lacZ*::P_{chb}-GFP, Kan^R; +8 SBS	A deletion of *lacZ* replaced with P_{chb}-GFP transcriptional fusion linked to Kan^R; SBS in promoter element was moved 8 bp upstream	This Study (CAK 216 / SAD 1462)
+8 SBS ΔCBP	Δ*lacZ*::P_{chb}-GFP, Kan^R; +8 SBS; ΔCBP::$Spec^R$	A deletion of *lacZ* replaced with P_{chb}-GFP transcriptional fusion linked to Kan^R; SBS in promoter element was moved 8 bp upstream; a deletion of CBP replaced with $Spec^R$	This Study (CAK 224 / SAD 1470)
-9 SBS	Δ*lacZ*::P_{chb}-GFP, Kan^R; -9 SBS	A deletion of *lacZ* replaced with P_{chb}-GFP transcriptional fusion linked to Kan^R; SBS in promoter	This Study (CAK 219 / SAD 1465)

		element was moved 9 bp downstream	
-9 SBS ΔCBP	Δ*lacZ*::P_{chb}-GFP, Kan^R; -9 SBS; ΔCBP::$Spec^R$	A deletion of *lacZ* replaced with P_{chb}-GFP transcriptional fusion linked to Kan^R; SBS in promoter element was moved 9 bp downstream; a deletion of CBP replaced with $Spec^R$	This Study (CAK 227 / SAD 1473)
+9 SBS	Δ*lacZ*::P_{chb}-GFP, Kan^R; +9 SBS	A deletion of *lacZ* replaced with P_{chb}-GFP transcriptional fusion linked to Kan^R; SBS in promoter element was moved 9 bp upstream	This Study (CAK 218 / SAD 1464)
+9 SBS ΔCBP	Δ*lacZ*::P_{chb}-GFP, Kan^R; +9 SBS; ΔCBP::$Spec^R$	A deletion of *lacZ* replaced with P_{chb}-GFP transcriptional fusion linked to Kan^R; SBS in promoter element was moved 9 bp upstream; a deletion of CBP replaced with $Spec^R$	This Study (CAK 226 / SAD 1472)
-10 SBS	Δ*lacZ*::P_{chb}-GFP, Kan^R; -10 SBS	A deletion of *lacZ* replaced with P_{chb}-GFP transcriptional fusion linked to Kan^R; SBS in promoter element was moved 10 bp downstream	This Study (CAK 200 / SAD 1456)
-10 SBS ΔCBP	Δ*lacZ*::P_{chb}-GFP, Kan^R; -10 SBS; ΔCBP::$Spec^R$	A deletion of *lacZ* replaced with P_{chb}-GFP transcriptional fusion linked to Kan^R; SBS in promoter element was moved 10 bp downstream; a deletion of CBP was replaced with $Spec^R$	This Study (CAK 201 / SAD 1457)
+10 SBS	Δ*lacZ*::P_{chb}-GFP, Kan^R; +10 SBS	A deletion of *lacZ* replaced with P_{chb}-GFP transcriptional fusion linked to Kan^R; SBS in promoter element was moved 10 bp upstream	This Study (CAK 202 / SAD 1458)
+10 SBS ΔCBP	Δ*lacZ*::P_{chb}-GFP, Kan^R; +10 SBS; ΔCBP::$Spec^R$	A deletion of *lacZ* replaced with P_{chb}-GFP transcriptional fusion linked to Kan^R; SBS in promoter element was moved 10 bp upstream; a deletion of CBP was replaced with $Spec^R$	This Study (CAK 203 / SAD 1459)
-11 SBS	Δ*lacZ*::P_{chb}-GFP, Kan^R; -11 SBS	A deletion of *lacZ* replaced with P_{chb}-GFP transcriptional fusion linked to Kan^R; SBS in promoter element was moved 11 bp downstream	This Study (CAK 221 / SAD 1467)
-11 SBS ΔCBP	Δ*lacZ*::P_{chb}-GFP, Kan^R; -11 SBS; ΔCBP::$Spec^R$	A deletion of *lacZ* replaced with P_{chb}-GFP transcriptional fusion linked to Kan^R; SBS in promoter element was moved 11 bp downstream; a deletion of CBP replaced with $Spec^R$	This Study (CAK 229 / SAD 1475)
+11 SBS	Δ*lacZ*::P_{chb}-GFP, Kan^R; +11 SBS	A deletion of *lacZ* replaced with P_{chb}-GFP transcriptional fusion linked to Kan^R; SBS in promoter	This Study (CAK 220 / SAD 1466)

		element was moved 11 bp upstream	
+11 SBS ΔCBP	Δ*lacZ*::P_{chb}-GFP, Kan^R; +11 SBS; ΔCBP::$Spec^R$	A deletion of *lacZ* replaced with P_{chb}-GFP transcriptional fusion linked to Kan^R; SBS in promoter element was moved 11 bp upstream; a deletion of CBP replaced with $Spec^R$	This Study (CAK 228 / SAD 1474)
-12 SBS	Δ*lacZ*::P_{chb}-GFP, Kan^R; -12 SBS	A deletion of *lacZ* replaced with P_{chb}-GFP transcriptional fusion linked to Kan^R; SBS in promoter element was moved 12 bp downstream	This Study (CAK 223 / SAD 1469)
-12 SBS ΔCBP	Δ*lacZ*::P_{chb}-GFP, Kan^R; -12 SBS; ΔCBP::$Spec^R$	A deletion of *lacZ* replaced with P_{chb}-GFP transcriptional fusion linked to Kan^R; SBS in promoter element was moved 12 bp downstream; a deletion of CBP replaced with $Spec^R$	This Study (CAK 231 / SAD 1477)
+12 SBS	Δ*lacZ*::P_{chb}-GFP, Kan^R; +12 SBS	A deletion of *lacZ* replaced with P_{chb}-GFP transcriptional fusion linked to Kan^R; SBS in promoter element was moved 12 bp upstream	This Study (CAK 222 / SAD 1468)
+12 SBS ΔCBP	Δ*lacZ*::P_{chb}-GFP, Kan^R; +12 SBS; ΔCBP::$Spec^R$	A deletion of *lacZ* replaced with P_{chb}-GFP transcriptional fusion linked to Kan^R; SBS in promoter element was moved 12 bp upstream; a deletion of CBP replaced with $Spec^R$	This Study (CAK 230 / SAD 1476)
Δ*lacZ*::P_{tac}-*slmA* T31A Δ*slmA*	Δ*lacZ*::P_{tac}-SlmA T31A $Spec^R$, Δ*slmA*::Tm^R, ΔVC1807::P_{chb}-*gfp* Kan^R	Overexpression construct for the indicated SlmA allele was integrated at the lacZ locus, while the P_{chb} reporter was integrated at the VC1807 locus, strain also has a deletion of *slmA*	This study (SAD1239)
Δ*lacZ*::P_{tac}-*slmA* T31A Δ*cbp* Δ*slmA*	Δ*lacZ*::P_{tac}-SlmA T31A $Spec^R$, Δ*slmA*:: Tm^R, Δ*cbp*::$Carb^R$, ΔVC1807::P_{chb}-*gfp*	Overexpression construct for the indicated SlmA allele was integrated at the lacZ locus, while the P_{chb} reporter was integrated at the VC1807 locus, strain also has a deletion of *slmA* and *cbp*	This study (SAD1242)
Δ*lacZ*::P_{tac}-*slmA* F63A Δ*slmA*	Δ*lacZ*::P_{tac}-SlmA F63A, Δ*slmA*::Tm^R, ΔVC1807::P_{chb}-*gfp*	Overexpression construct for the indicated SlmA allele was integrated at the lacZ locus, while the P_{chb} reporter was integrated at the VC1807 locus, strain also has a deletion of *slmA*	This study (SAD1240)
Δ*lacZ*::P_{tac}-*slmA* F63A Δ*cbp* Δ*slmA*	Δ*lacZ*::P_{tac}-SlmA F63A, Δ*slmA*::Tm^R, Δ*cbp*::$Carb^R$, ΔVC1807::P_{chb}-*gfp*	Overexpression construct for the indicated SlmA allele was integrated at the lacZ locus, while the P_{chb} reporter was integrated at the VC1807 locus, strain also has a deletion of *slmA* and *cbp*	This study (SAD1243)

Δ*lacZ*::P_{tac}-*slmA* R71D Δ*slmA*	Δ*lacZ*::P_{tac}-SlmA R71D, Δ*slmA*::Tm^R, ΔVC1807::P_{chb}-*gfp*	Overexpression construct for the indicated SlmA allele was integrated at the lacZ locus, while the P_{chb} reporter was integrated at the VC1807 locus, strain also has a deletion of *slmA*	This study (SAD1241)
Δ*lacZ*::P_{tac}-*slmA* R71D Δ*cbp* Δ*slmA*	Δ*lacZ*::P_{tac}-SlmA R71D, Δ*slmA*::Tm^R, Δ*cbp*::$Carb^R$, ΔVC1807::P_{chb}-*gfp*	Overexpression construct for the indicated SlmA allele was integrated at the lacZ locus, while the P_{chb} reporter was integrated at the VC1807 locus, strain also has a deletion of *slmA* and *cbp*	This study (SAD1244)
P_{chb} 112 bp *lacZ* swap-gfp	Δ*lacZ*::P_{chb}-*gfp* 112 bp swap Kan^R	Δ*lacZ*::P_{chb}-*gfp* with nucleotides 612-724 swapped with an intergenic region from the *lacZ* gene linked to Kan^R	This Study (CAK 337 / SAD 1482)
P_{chb} 112 bp *lacZ* swap-gfp Δ*cbp*	Δ*lacZ*::P_{chb}-*gfp* 112 bp swap Kan^R, ΔCBP::$Spec^R$	Δ*lacZ*::P_{chb}-*gfp* with nucleotides 612-724 swapped with an intergenic region from the *lacZ* gene linked to Kan^R; deletion of CBP replaced with $Spec^R$	This Study (CAK 338 / SAD 1483)
Δ*rpoS* Δ*cbp*	P_{chb}-*gfp,* Kan^R; Δ*rpoS*::$Spec^R$; Δ*cbp*::Tm^R	A deletion of *lacZ* replaced with P_{chb}-GFP transcriptional fusion linked to Kan^R; a deletion of *cbp* was replaced with Tm^R and a deletion of *rpoS* replaced with $Spec^R$	This Study (CAH 432 / SAD 1486)
Δ*rpoN* Δ*cbp*	P_{chb}-*gfp,* Kan^R; Δ*rpoN*::$Spec^R$; Δ*cbp*::Tm^R	A deletion of *lacZ* replaced with P_{chb}-GFP transcriptional fusion linked to Kan^R; a deletion of *cbp* was replaced with Tm^R and a deletion of *rpoN* replaced with $Spec^R$	This Study (CAH 433 / SAD 1487)
Δ*rpoF* Δ*cbp*	P_{chb}-*gfp,* Kan^R; Δ*rpoF*::$Spec^R$; Δ*cbp*::Tm^R	A deletion of *lacZ* replaced with P_{chb}-GFP transcriptional fusion linked to Kan^R; a deletion of *cbp* was replaced with Tm^R and a deletion of *rpoF* replaced with $Spec^R$	This Study (CAH 434 / SAD 1488)
Δ*rpoH* Δ*cbp*	P_{chb}-*gfp,* Kan^R; Δ*rpoH*::$Spec^R$; Δ*cbp*::Tm^R	A deletion of *lacZ* replaced with P_{chb}-GFP transcriptional fusion linked to Kan^R; a deletion of *cbp* was replaced with Tm^R and a deletion of *rpoH* replaced with $Spec^R$	This Study (CAH 435 / SAD 1489)
Δ*rpoE* Δ*cbp*	P_{chb}-*gfp,* Kan^R; Δ*rpoE*::$Spec^R$; Δ*cbp*::Tm^R	A deletion of *lacZ* replaced with P_{chb}-GFP transcriptional fusion linked to Kan^R; a deletion of *cbp* was replaced with Tm^R and a deletion of *rpoE* replaced with $Spec^R$	This Study (CAH 436 / SAD 1490)
Ec SBS Ec SlmA	Δ*lacZ*::P_{chb}-GFP with Ec SBS, Kan^R; Ec SlmA; ΔVC1807::$Spec^R$	CAK 168 parent with the native copy of SlmA replaced with Ec SlmA; a deletion of VC1807 was replaced with $Spec^R$	This Study (CAK 393 / SAD1608)

Ec SBS Ec SlmA Δ*cbp*	Δ*lacZ*::P_{chb}-GFP with Ec SBS, Kan^R; Ec SlmA; ΔCBP::$Spec^R$;	CAK 168 parent with the native copy of SlmA replaced with Ec SlmA; a deletion of cbp was replace with $Spec^R$	This Study (CAK 394 / SAD1609)
P_{tac}-SlmA	Δ*lacZ*::P_{chb}-GFP, Kan^R; ΔVC1807::P_{tac}-SlmA-3x FLAG, $Spec^R$	CAK 007 parent with a deletion of VC1807 replaced with a SlmA-3x FLAG fusion linked to the IPTG-inducible P_{tac} promoter; P_{tac} construct is linked to $Spec^R$	This Study (CAK 371 / SAD1614)
P_{tac}-SlmA ΔCBP	Δ*lacZ*::P_{chb}-GFP, Kan^R; ΔVC1807::P_{tac}-SlmA-3x FLAG, $Spec^R$; ΔCBP::Tm^R	CAK 007 parent with a deletion of VC1807 replaced with a SlmA-3x FLAG fusion linked to the IPTG-inducible P_{tac} promoter; P_{tac} construct is linked to $Spec^R$; a deletion of *cbp* was replaced with Tm^R	This Study (CAK 372 / SAD1615)
P_{tac}-SlmA F63A	Δ*lacZ*::P_{chb}-GFP, Kan^R; ΔVC1807::P_{tac}-SlmA F63A-3x FLAG, $Spec^R$	Native copy of SlmA WT replaced with SlmA F63A; deletion of VC1807 replaced with a SlmA-3x FLAG fusion linked to the IPTG-inducible P_{tac} promoter; P_{tac} construct is linked to $Spec^R$	This Study (CAK 417 / SAD1616)
P_{tac}-SlmA F63A ΔCBP	Δ*lacZ*::P_{chb}-GFP, Kan^R; ΔVC1807::P_{tac}-SlmA F63A-3x FLAG, $Spec^R$; ΔCBP::$Carb^R$	Native copy of SlmA WT replaced with SlmA F63A; deletion of VC1807 replaced with a SlmA-3x FLAG fusion linked to the IPTG-inducible P_{tac} promoter; P_{tac} construct is linked to $Spec^R$; a deletion of *cbp* was replaced with $Carb^R$	This Study (CAK 418 / SAD1617)
P_{tac}-ChiS	Δ*lacZ*::P_{chb}-GFP, Kan^R; ΔVCA0692::Ptac-ChiS, Tm^R	CAK 007 parent with a deletion of VCA0692 replaced with ChiS linked to the IPTG-inducible P_{tac} promoter; P_{tac} construct is linked to Tm^R	This Study (CAK 409 / SAD1619)
P_{tac}-ChiS ΔCBP	Δ*lacZ*::P_{chb}-GFP, Kan^R; ΔVCA0692::Ptac-ChiS, Tm^R; ΔCBP::$Carb^R$	CAK 007 parent with a deletion of VCA0692 replaced with ChiS linked to the IPTG-inducible P_{tac} promoter; P_{tac} construct is linked to Tm^R; a deletion of *cbp* was replaced with $Carb^R$	This Study (CAK 410 / SAD1620)
P_{tac}-SlmA P_{tac}-ChiS	Δ*lacZ*::P_{chb}-GFP, Kan^R; ΔVC1807::P_{tac}-SlmA-3x FLAG, $Spec^R$; ΔVCA0692::Ptac-ChiS, Tm^R	CAK 371 parent with a deletion of VCA0692 replaced with ChiS linked to the IPTG-inducible P_{tac} promoter; P_{tac}-ChiS construct is linked to Tm^R	This Study (CAK 415 / SAD1621)
P_{tac}-SlmA P_{tac}-ChiS ΔCBP	Δ*lacZ*::P_{chb}-GFP, Kan^R; ΔVC1807::P_{tac}-SlmA-3x FLAG, $Spec^R$; ΔVCA0692::Ptac-ChiS, Tm^R; ΔCBP::$Carb^R$	CAK 371 parent with a deletion of VCA0692 replaced with ChiS linked to the IPTG-inducible P_{tac} promoter; P_{tac}-ChiS construct is linked to Tm^R; a deletion of *cbp* was replaced with $Carb^R$	This Study (CAK 416 / SAD1622)
P_{tac}-SlmA F63A P_{tac}-ChiS	Δ*lacZ*::P_{chb}-GFP, Kan^R; ΔVC1807::P_{tac}-SlmA F63A-3x FLAG, $Spec^R$;	CAK 417 parent with a deletion of VCA0692 replaced with ChiS linked to the IPTG-inducible P_{tac}	This Study (CAK 419 / SAD1623)

	ΔVCA0692::Ptac-ChiS, Tm^R	promoter; P_{tac}-ChiS construct is linked to Tm^R	
P_{tac}-SlmA F63A P_{tac}-ChiS ΔCBP	Δ*lacZ*::P_{chb}-GFP, Kan^R; ΔVC1807::P_{tac}-SlmA F63A-3x FLAG, $Spec^R$; ΔVCA0692::Ptac-ChiS, Tm^R; ΔCBP::$Carb^R$	CAK 417 parent with a deletion of VCA0692 replaced with ChiS linked to the IPTG-inducible P_{tac} promoter; P_{tac}-ChiS construct is linked to Tm^R; a deletion of *cbp* was replaced with $Carb^R$	This Study (CAK 420 / SAD1624)
**Strains Used for Ec SBS-GFP Repression Reporter Assays**			
WT SlmA	ΔVC1807::Ec SBS-GFP, $Spec^R$	A deletion of VC1807 replaced with consensus sequence of *E. coli* SlmA Binding Sites flanked by -10 and -35 RNA polymerase binding sites fused to GFP and linked to $Spec^R$	This Study (CAK 135 / SAD 1427)
ΔSlmA	ΔVC1807::Ec SBS-GFP, $Spec^R$; Δ*slmA*::Tm^R	CAK 135 parent with a deletion of *slmA* replaced with Tm^R	This Study (CAK 136 / SAD 1428)
SlmA T31A	ΔVC1807::Ec SBS-GFP, $Spec^R$; SlmA T31A; Δ*lacZ* LPQEN::Kan^R	CAK 136 parent with native *slmA* replaced with *slmA* T31A; *lacZ* linked to Kan^R	This Study (CAK 195 / SAD 1454)
SlmA E43A	ΔVC1807::Ec SBS-GFP, $Spec^R$; SlmA E43A; Δ*lacZ* LPQEN::Kan^R	CAK 136 parent with native *slmA* replaced with *slmA* E43A; *lacZ* linked to Kan^R	This Study (CAK 244 / SAD 1478)
SlmA E43K	ΔVC1807::Ec SBS-GFP, $Spec^R$; SlmA E43K; Δ*lacZ* LPQEN::Kan^R	CAK 136 parent with native *slmA* replaced with *slmA* E43K; *lacZ* linked to Kan^R	This Study (CAK 212 / SAD 1461)
SlmA F63A	ΔVC1807::Ec SBS-GFP, $Spec^R$; SlmA F63A; Δ*lacZ* LPQEN::Kan^R	CAK 136 parent with native *slmA* replaced with *slmA* F63A; *lacZ* linked to Kan^R	This Study (CAK 187 / SAD 1449)
SlmA R71D	ΔVC1807::Ec SBS-GFP, $Spec^R$; SlmA R71D; Δ*lacZ* LPQEN::Kan^R	CAK 136 parent with native *slmA* replaced with *slmA* R71D; *lacZ* linked to Kan^R	This Study (CAK 188 / SAD 1450)
SlmA R173E	ΔVC1807::Ec SBS-GFP, $Spec^R$; SlmA R173E; Δ*lacZ* LPQEN::Kan^R	CAK 136 parent with native *slmA* replaced with *slmA* R173E; *lacZ* linked to Kan^R	This Study (CAK 189 / SAD 1451)
**Strains Used for Microscopy**			
WT SlmA	Δ*lacZ*::P_{tac}-SlmA, $Spec^R$	*lacZ* replaced with SlmA WT under the control of an IPTG inducible P_{tac} promoter (derived from a $Spec^R$ Tn10 fragment containing LacI, $Spec^R$, and an outward reading P_{tac} promoter)	This Study (CAK 137 / SAD 1429)
SlmA T31A	Δ*lacZ*::P_{tac}-SlmA, $Spec^R$	*lacZ* replaced with SlmA T31A under the control of an IPTG inducible P_{tac} promoter (derived from a $Spec^R$ Tn10 fragment containing LacI, $Spec^R$, and an outward reading P_{tac} promoter)	This Study (CAK 151 / SAD 1432)
SlmA E43A	Δ*lacZ*::P_{tac}-SlmA, $Spec^R$	*lacZ* replaced with SlmA E43A under the control of an IPTG inducible P_{tac} promoter (derived from a $Spec^R$ Tn10 fragment containing LacI, $Spec^R$, and an outward reading P_{tac} promoter)	This Study (CAK 152 / SAD 1433)

SlmA E43K	Δ*lacZ*::P_{tac}-SlmA, $Spec^R$	*lacZ* replaced with SlmA E43K under the control of an IPTG inducible P_{tac} promoter (derived from a $Spec^R$ Tn10 fragment containing LacI, $Spec^R$, and an outward reading P_{tac} promoter)	This Study (CAK 341 / SAD 1484)
SlmA F63A	Δ*lacZ*::P_{tac}-SlmA, $Spec^R$	*lacZ* replaced with SlmA F63A under the control of an IPTG inducible P_{tac} promoter (derived from a $Spec^R$ Tn10 fragment containing LacI, $Spec^R$, and an outward reading P_{tac} promoter)	This Study (CAK 153 / SAD 1434)
SlmA R71D	Δ*lacZ*::P_{tac}-SlmA, $Spec^R$	*lacZ* replaced with SlmA R71D under the control of an IPTG inducible P_{tac} promoter (derived from a $Spec^R$ Tn10 fragment containing LacI, $Spec^R$, and an outward reading P_{tac} promoter)	This Study (CAK 154 / SAD 1435)
SlmA R173E	Δ*lacZ*::P_{tac}-SlmA, $Spec^R$	*lacZ* replaced with SlmA R173E under the control of an IPTG inducible P_{tac} promoter (derived from a $Spec^R$ Tn10 fragment containing LacI, $Spec^R$, and an outward reading P_{tac} promoter)	This Study (CAK 342 / SAD 1485)
**Strains Used for SlmA Overexpression and Purification**			
	E. coli BL21-DE3 harboring pHisTev w / WT SlmA	WT SlmA ovexpression strain – SlmA cloned into NdeI / BamHI sites	This Study (CAK 110 / SAD 1414)
	E. coli BL21-DE3 harboring pHisTev + SlmA T31A	SlmA T31A ovexpression strain– vector generated using single primer site directed mutagenesis	This Study (CAK 161 / SAD 1436)
	E. coli BL21-DE3 harboring pHisTev + SlmA E43A	SlmA E43A ovexpression strain– vector generated using single primer site directed mutagenesis	This Study (CAK 162 / SAD 1437)
	E. coli BL21-DE3 harboring pHisTev + SlmA E43K	SlmA E43K ovexpression strain– vector generated using single primer site directed mutagenesis	This study (CAK 268 / SAD 1479)
**Strains Used for FLAG Western Blots**			
SlmA WT-3x FLAG	Δ*lacZ*::P_{chb}-GFP, Kan^R; SlmA WT-3x FLAG; ΔCBP::$Spec^R$	CAK 024 parent with SlmA WT-3x FLAG knocked back in at native locus; deletion of *cbp* replaced with $Spec^R$	This study (CAK 426 / SAD 1601)
SlmA T31A-3x FLAG	Δ*lacZ*::P_{chb}-GFP, Kan^R; SlmA T31A-3x FLAG; ΔCBP::$Spec^R$	CAK 024 parent with SlmA T31A-3x FLAG knocked back in at native locus; deletion of *cbp* replaced with $Spec^R$	This study (CAK 380 / SAD 1602)
SlmA E43A-3x FLAG	Δ*lacZ*::P_{chb}-GFP, Kan^R; SlmA E43A-3x FLAG; ΔCBP::$Spec^R$	CAK 024 parent with SlmA E43A-3x FLAG knocked back in at native locus; deletion of *cbp* replaced with $Spec^R$	This study (CAK 382 / SAD 1603)
SlmA E43K-3x FLAG	Δ*lacZ*::P_{chb}-GFP, Kan^R; SlmA E43K-3x FLAG; ΔCBP::$Spec^R$	CAK 024 parent with SlmA E43K-3x FLAG knocked back in at native locus; deletion of *cbp* replaced with $Spec^R$	This study (CAK 384 / SAD 1604)

SlmA F63A-3x FLAG	Δ*lacZ*::P_{chb}-GFP, Kan^R; SlmA F63A-3x FLAG; ΔCBP::$Spec^R$	CAK 024 parent with SlmA F63A-3x FLAG knocked back in at native locus; deletion of *cbp* replaced with $Spec^R$	This study (CAK 386 / SAD 1605)
SlmA R71D-3x FLAG	Δ*lacZ*::P_{chb}-GFP, Kan^R; SlmA R71D-3x FLAG; ΔCBP::$Spec^R$	CAK 024 parent with SlmA R71D-3x FLAG knocked back in at native locus; deletion of *cbp* replaced with $Spec^R$	This study (CAK 388 / SAD 1606)
SlmA R173E-3x FLAG	Δ*lacZ*::P_{chb}-GFP, Kan^R; SlmA R173E-3x FLAG; ΔCBP::$Spec^R$	CAK 024 parent with SlmA R173E-3x FLAG knocked back in at native locus; deletion of *cbp* replaced with $Spec^R$	This study (CAK 390 / SAD 1607)
Ptac-ChiS-FLAG	Δ*lacZ*::P_{chb}-GFP, Kan^R; ΔVCA0692::Ptac-ChiS-1x FLAG, Tm^R; ΔChiS::$Spec^R$; ΔCBP::$Carb^R$	CAK 007 parent with a deletion of VCA0692 replaced with a ChiS-1x FLAG fusion linked to the IPTG-inducible P_{tac} promoter; P_{tac}-ChiS construct is linked to Tm^R; deletion of *chiS* replaced with $Spec^R$ and a deletion of *cbp* replaced with $Carb^R$	This study (CAK 406 / SAD 1618)
ChiS-FLAG	Δ*lacZ*::P_{chb}-GFP, Kan^R; ΔChiS::ChiS-1x FLAG; ΔCBP::Tm^R	CAK 007 parent with native ChiS replaced with a ChiS-1x FLAG fusion; deletion of CBP replaced with Tm^R	This study (CAK 374 / SAD 1625)
**Strains Used for mCherry assays**			
WT	Δ*lacZ*::P_{chb}-mCherry, Kan^R	A deletion of *lacZ* replaced with P_{chb}-mCherry transcriptional fusion linked to Kan^R	This study (CAK 346 / SAD 1610)
Δcbp	Δ*lacZ*::P_{chb}-mCherry, Kan^R; ΔCBP::$Spec^R$	CAK 346 parent with a deletion of *cbp* replaced with $Spec^R$	This study (CAK 347 / SAD 1611)
SlmA-3x FLAG	Δ*lacZ*::P_{chb}-mCherry, Kan^R; ΔSlmA::SlmA-3x FLAG; ΔVC1807::$Spec^R$	CAK 346 parent with the native copy of SlmA replaced with SlmA-3x FLAG; deletion of VC1807 replaced with $Spec^R$	This study (CAK 358 / SAD 1612)
SlmA-3x FLAG Δcbp	Δ*lacZ*::P_{chb}-mCherry, Kan^R; ΔSlmA::SlmA-3x FLAG; ΔCBP::$Spec^R$	A deletion of *lacZ* replaced with P_{chb}-mCherry transcriptional fusion linked to Kan^R; native copy of SlmA replaced with SlmA-3x FLAG; deletion of CBP replaced with $Spec^R$	This study (CAK 359 / SAD 1613)
