# Supplementary material for: The nucleoid occlusion protein SlmA is a direct transcriptional activator of chitobiose utilization in Vibrio cholerae
Source: PLoS Genet. 2017 Jul 6;13(7):e1006877. doi: 10.1371/journal.pgen.1006877 (PMC5519180; doi:10.1371/journal.pgen.1006877)
Supplement: S2 Table — (PDF) [file pgen.1006877.s015.pdf]

Table S2 – *Primers used in this study*

Primer Name	Primer Sequence (5'→3')	Description
**Primers for SOE Deletions**		
ABD 796	TTAGAATCTGCGCCAGAAGCG	ΔCBP F1
ABD 797	gtcgacggatccccggaatCATAGCTGTTCCTTACTAGTTGC	ΔCBP R1
ABD 798	gaagcagctccagcctacaGTACTGGATCTGAAACCAGTTAAG	ΔCBP F2
ABD 799	GTATTGCGGAATGACCAGCATG	ΔCBP R2
CAH 009	TGATGTTCATGCTCTGCACC	ΔSlmA F1
CAH 010	gtcgacggatccccggaatCATGCCCCGTTTTTCCTTTTG	ΔSlmA R1
CAH 011	gaagcagctccagcctacaATTAAGTGAGTACAATGAGTCAAG	ΔSlmA F2
CAH 012	GAAGGCATCGTTGTTAGATTGA	ΔSlmA R2
ABD 767	TTAATTTGGATCCCTGCGACACTC	ΔChiS F1
ABD 768	gtcgacggatccccggaatCAAAAAACGTGAGGAGAATGCC	ΔChiS R1
ABD 769	gaagcagctccagcctacaTTCTTGAGCATTGCAAAGAAGC	ΔChiS F2
ABD 770	CTGGAACGAATGAAGAAGTCCAG	ΔChiS R2
ABD 927	GCAGAGAAAGGGTATCATTACTGG	ΔVC0995 F1
ABD 928	GcTAATTCAGTTTAAGCGGCCATCTTAAGTTCCCCCTATAG GATTTTTG	ΔVC0995 R1
ABD 929	ATGGCCGCTTAAACTGAATTAgCACATCAGGTGCTTTAGGC CAATTTG	ΔVC0995 F2
ABD 930	TACTCTCGTTTTTCGGCTTACTC	ΔVC0995 R2
BBC 081	AAGCAAGTTCACGTTTGCCG	ΔVC0618-19 F1
BBC 082	gtcgacggatccccggaatCATAACTTACACCTTACTCACCCAG	ΔVC0618-19 R1
BBC 083	gaagcagctccagcctacaGGAGATAAATAATCATGACTACGCC	ΔVC0618-19 F2
BBC 084	TAAAGTTCGCAACACGCC	ΔVC0618-19 R2
BBC 285	TCACTCTGCGTTTCTCATTGG	Δ*rpoS* F1
BBC 286	gtcgacggatccccggaatCATAGCGGCCTCCCCCTGG	Δ*rpoS* R1
BBC 287	gaagcagctccagcctacaTAATTTTTCCAGACTCATCCAAAAC	Δ*rpoS* F2
BBC 288	AAAACCCAGACATTCTGCTG	Δ*rpoS* R2
CAH 050	CTCTAATTCCGGAGAATCATCAG	Δ*rpoN* F1
CAH 051	gtcgacggatccccggaatCATGCAGTAATGGATGCCTTG	Δ*rpoN* R1
CAH 052	gaagcagctccagcctacaCTATAGGCCTAAACTAGAGAAGG	Δ*rpoN* F2
CAH 053	GAGAGTCGCTTCAAACATGTTC	Δ*rpoN* R2
CAH 095	CTGCACGTATTGTTGAACAAGAG	Δ*rpoF* F1
CAH 096	gtcgacggatccccggaatCATTCAATTCCTCATCATTCTCTG	Δ*rpoF* R1
CAH 097	gaagcagctccagcctacaTGATTTCACATTGCGTAACAGC	Δ*rpoF* F2
CAH 098	GATCAGGAGCAGTACTATGAC	Δ*rpoF* R2
CAH 100	CGATATTCGCACCATAGTGC	Δ*rpoH* F1
CAH 101	gtcgacggatccccggaatGTATTGATCGTATGTAAGCGC	Δ*rpoH* R1
CAH 102	gaagcagctccagcctacaCGGAATGACTAAATATCACTGATC	Δ*rpoH* F2
CAH 103	CCATGGTAGAGTCAATATCGATC	Δ*rpoH* R2
CAH 105	GAGATGCGTGAGCATCAGTTC	Δ*rpoE* F1
CAH 106	gtcgacggatccccggaatCATTCGAGCGGTCACTCCTATTG	Δ*rpoE* R1
CAH 107	gaagcagctccagcctacaCTTCTGTAACGCAAATTCCG	Δ*rpoE* F2
CAH 108	GAGCAACGCACAGCATCAATC	Δ*rpoE* R2
BBC 717	AAATAGATTTGGTGACTTTACCTCC	ΔVC1807 F1
ABD 340	gtcgacggatccccggaatACGTTTCATTAGTCACCTCTATTGTT AACTTGTTC	ΔVC1807 R1

ABD 341	gaagcagctccagcctacaTAGTCGAAAATAAAAAAAAGAGGCTC GCCTC	ΔVC1807 F2
BBC 718	CTTTACGCCTGATTGTCTACAC	ΔVC1807 R2
ABD 123	ATTCCGGGGATCCGTCGAC	Kan^R, $Spec^R$, Amp^R, or Tm^R cassette F
ABD 124	TGTAGGCTGGAGCTGCTTC	Kan^R, $Spec^R$, Amp^R, or Tm^R cassette R
**Primers for Colony PCR**		
ABD 725	GAAGCAGCTCCAGCCTACA	F oligo to detect all SOE mutants
CAH 023	GGTGATTTCCAGATTGAGTGC	Δ*slmA* detect R (401 bp)
BBC 085	AACTTCCCAACCCTTTGG	ΔVC0618-19 detect R (780 bp)
BBC 289	CAGCTGAGCTTAATTCCTG	Δ*rpoS* detect R (225 bp)
CAH 054	GTCGAACCAATTTATCCACC	Δ*rpoN* detect R (254 bp)
CAH 099	CGCTAGAGTCAAACTACTTAGAG	Δ*rpoF* detect R (176 bp)
CAH 104	CAGGTGCTTCAGTTCTTCGTC	Δ*rpoH* detect R (240 bp)
CAH 109	GCTTCCAGCAAGAGGAATGG	Δ*rpoE* detect R (447 bp)
CKP 043	TCACTTAATCTGTGCACTTAGCAG	R oligo to detect all SlmA point mutants
CKP 045	GAAGGGGCTTCACGCATtg	SlmA T31A F (520 bp)
CKP 097	AAGCAAGTTGGCGTGTCCac	SlmA E43A F (487 bp)
CKP 119	CGCTAAGCAAGTTGGCGTGTCta	SlmA E43K F (487 bp)
CKP 042	TTTGAAGGCTTAATTGAGgcg	SlmA F63A F (420 bp)
CKP 044	GAAGAATCCTTGATGTCGga	SlmA R71D F (400 bp)
BBC 895	TTGAATCGCTTTGTGgaa	SlmA R173E F (90 bp)
ABD 769	gaagcagctccagcctacaTTCTTGAGCATTGCAAAGAAGC	Scrambled SBS detect F
CKP 053	GTTTAGCTAGGTTtaaTAGTGTTTctg	1st conserved SBS site scrambled R
CKP 054	GGTACGGTTTAGCTAGGTTtaa	2nd conserved SBS site scrambled R
ABD 797	gtcgacggatccccggaatCATAGCTGTTCCTTACTAGTTGC	ΔSBS detect R
CKP 052	AGCCATTATCAAACAAGTAGCTAAACCGTA	ΔSBS detect F
**Primers for Transcriptional Reporter Constructs**		
ABD332	GGCTGAACGTGGTTGTCGAAAATGAC	Δ*lacZ* F1 (Up Arm)
BBC219	GTTTATTTTTGTCGACTGTACAGCGTTTAAATAGAGGTCG ATATTGACCC	Δ*lacZ* R1 (Up Arm)
BBC218	CGCTGTACAGTCGACAAAAATAAAC	Kan^R F (Middle Arm)
BBC262	TACCGAGGACGCGAAGCTG	Kan^R R (Middle Arm)
BBC266	CAGCTTCGCGTCCTCGGTAGAATAAAGCAATCCGCAAGCG	P_{chb} F (Middle Arm)
BBC267	CCCGGGATCCTGTGTGAAATTGAGTTGCTTTCATTTCACTA ATGG	P_{chb} R (Middle Arm)
BBC252	CAATTTCACACAGGATCCCGGGAGGAGGTAACGTAATGCG TAAAGGAGAAGAAC	GFP F (Middle Arm)
BBC254	tgtaggctggagctgcttcTTAGTTGTATAGTTCATCCATGCC	GFP R (Middle Arm)
ABD255	gaagcagctccagcctacaCCACAATAAGCCAGAGAGCCTTAAG	Δ*lacZ* F2 (Down Arm)
ABD256	CCCAAATACGGCAACTTGGCG	Δ*lacZ* R2 (Down Arm)
BBC817	ttgagtaagtgagcgctcacttactataatgtgtggAATTGTGAGCGGAT AACAATTTCA	Synthetic SBS-GFP DNA biding reporter F2
BBC821	ccacacattatagtaagtgagcgctcacttactcaaCTCATTAGGCACCC CAGGC	Synthetic SBS-GFP DNA biding reporter R1
CKP210	TGACCATTTAGAGATGCTAGGTTTGTTAGTGTTTACTTAC	P_{chb} 612-724 lacZ swap R1

CKP212	ACATTCCTGTACCGAACGGGAATTGCAATTGATAAATTTC	P_{chb} 612-724 lacZ swap F2
CKP213	ACTAACAAACCTAGCATCTCTAAATGGTCAGTGGCG	112 bp *lacZ* intergenic F
CKP215	AATTGCAATTCCCGTTCGGTACAGGAATGTGCGCCCAAG	112 bp *lacZ* intergenic R
**Primers for SlmA Mutants**		
CKP 040	GAAGGGGCTTCACGCATCgCgACCGCAAAACTCGCTAAGCAAG	SlmA T31A F2
CKP 041	GCGAGTTTTGCGGTcGcGATGCGTGAAGCCCCTTCATTG	SlmA T31A R1
CKP 095	TGGCGTGTCCGcAGCCGCGCTGTATCGCCATTTCCCGAGC	SlmA E43A F2
CKP 096	TGGCGATACAGCGCGGCTgCGGACACGCCAACTTGCTTAG	SlmA E43A R1
CKP 117	TGGCGTGTCCaAAGCCGCGCTGTATCGCCATTTCCCGAGC	SlmA E43K F2
CKP 118	TGGCGATACAGCGCGGCTTtGGACACGCCAACTTGCTTAG	SlmA E43K R1
CKP 036	TGAAGGCTTAATTGAGgcgATTGAAGAATCCTTGATGTCG	SlmA F63A F2
CKP 037	AGGATTCTTCAATcgcCTCAATTAAGCCTTCAAACATACG	SlmA F63A R1
CKP 038	GAATCCTTGATGTCGgaTATCAACCGCATCTTTGATGAAG	SlmA R71D F2
CKP 039	GATGCGGTTGATAtcCGACATCAAGGATTCTTCAAT	SlmA R71D R1
BBC 894	GGCAGTTTGAATCGCTTTGTGgaaTCTGATTTCAAATATCTGCC	SlmA R173E F2
BBC 893	GGCAGATATTTGAAATCAGAttcCACAAAGCGATTCAAACTGCC	SlmA R173E R1
CAH0009	TGATGTTCATGCTCTGCACC	Replace Vc *slmA* with Ec *slmA* F1
BBC768	GCCCCGTTTTTCCTTTTGG	Replace Vc *slmA* with Ec *slmA* R1
BBC770	ccaaaaggaaaaacggggcATGGCAGAAAAACAAACTGC	Replace Vc *slmA* with Ec *slmA* (middle) F
BBC771	gctgtatttatcttgactcattgtacTTcAtTGtAACTGTGCCGCAATTAG	Replace Vc *slmA* with Ec *slmA* (middle) R
BBC769	GTACAATGAGTCAAGATAAATACAGC	Replace Vc *slmA* with Ec *slmA* F2
CAH0012	GAAGGCATCGTTGTTAGATTGA	Replace Vc *slmA* with Ec *slmA* R2
**Primers for P_{chb} Modifications**		
CKP 102	gtaagtgagcgctcacttacCTAGCTAAACCGTACCCGTTTTG	P_{chb} *E. coli* SBS F
CKP 101	gtaagtgagcgctcacttacTTATTTGGCCTTGTTTGATAATGG	P_{chb} *E. coli* SBS R
CKP 049	ATTATCAAACAAGGCCAAATAAGTAcagAAACACTAttaAACCTAGCTAAACCGTACCCG	P_{chb} Scrambled SBS F
CKP 048	GGGTACGGTTTAGCTAGGTTtaaTAGTGTTTctgTACTTATTTGGCCTTGTTTGATAATG	P_{chb} Scrambled SBS R
BBC 266	CAGCTTCGCGTCCTCGGTAGAATAAAGCAATCCGCAAGCG	P_{chb} P1 only and P1+P2 F
BBC 609	cccgggatcctgtgtgaaattgCCTAGCGGCAATTCAAGTTGC	P_{chb} P1 only R
BBC 608	cccgggatcctgtgtgaaattgCTGAGTTATATTTGCGAGATCTCGC	P_{chb} P1+P2 R
BBC 606	cagcttcgcgtcctcggtaGAGATTGCGAAGGGAGTCAC	P_{chb} P2 only, P2+SBS, P2+SBS more space, P2+P3, P3 only more space, Δhairpin F
CKP 001	cccgggatcctgtgtgaaattgCTTATTTGGCCTTGTTTGATAATG	P_{chb} P2 only R
CKP 071	cccgggatcctgtgtgaaattgGTGATTCAACTCGCAAAACGGG	P_{chb} P2+SBS R
CKP 072	cccgggatcctgtgtgaaattgCTTTGGCAGGAGTAAGAAAACACCTAG	P_{chb} P2+SBS more space and P2+P3 internal region R
BBC 267	CCCGGGATCCTGTGTGAAATTGAGTTGCTTTCATTTCACTAATGG	P_{chb} P2+P3 and P3 only R
CKP 073	cagcttcgcgtcctcggtaCAGGCTAGTGAGCGAGATCT	P_{chb} P2+P3 internal region and P3 only more space F
BBC 607	cagcttcgcgtcctcggtaTACAGGCCACTCATGACTCC	P_{chb} P3 only F
CKP 084	cccgggatcctgtgtgaaattgGACCTACCTCATCACTTTTACCC	P_{chb} Δhairpin R

CKP 106	AACAAGGCCAAAGTAAGTAAACACTAACAAACtaaCTAGCT AAACCGTACCCG	P_{chb} -3 SBS F
CKP 105	ttagtttgttagtgtttacttacTTTGGCCTTGTTTGATAATGGC	P_{chb} -3 SBS R
CKP 108	GCCAAATAActaGTAAGTAAACACTAACAAACGCTAAACCG TACCCGTTTTG	P_{chb} +3 SBS F
CKP 107	AGCGTTTGTTAGTGTTTACTTACtagTTATTTGGCCTTGTT TGATAATGG	P_{chb} +3 SBS R
CKP 104	GGCCAGTAAGTAAACACTAACAAACaataaCTAGCTAAACCG TACCCGTTTTG	P_{chb} -5 SBS F
CKP 103	GCTAGttattGTTTGTTAGTGTTTACTTACTGGCCTTGTTTGA TAATGGC	P_{chb} -5 SBS R
CKP 110	GGCCAAATAActagcGTAAGTAAACACTAACAAACTAAACCG TACCCGTTTTGCG	P_{chb} +5 SBS F
CKP 109	GGTTTAGTTTGTTAGTGTTTACTTACgctagTTATTTGGCCT TGTTTGATAATGG	P_{chb} +5 SBS R
CKP 136	GTAAGTAAACACTAACAAACccaaataaCTAGCTAAACCGTA CCCG	P_{chb} -8 SBS F
CKP 135	ttatttggGTTTGTTAGTGTTTACTTACCCTTGTTTGATAATG GCTCTTTGCC	P_{chb} -8 SBS R
CKP 138	ctagctaaGTAAGTAAACACTAACAAACACCGTACCCGTTTTG CGAGTTGAATC	P_{chb} +8 SBS F
CKP 137	GTTTGTTAGTGTTTACTTACttagctagTTATTTGGCCTTGTT TGATAATGGC	P_{chb} +8 SBS R
CKP 132	GTAAGTAAACACTAACAAACgccaaataaCTAGCTAAACCGT ACCCG	P_{chb} -9 SBS F
CKP 131	ttatttggcGTTTGTTAGTGTTTACTTACCTTGTTTGATAATG GCTCTTTGCC	P_{chb} -9 SBS R
CKP 134	ctagctaaaGTAAGTAAACACTAACAAACCCGTACCCGTTTTG CGAGTTGAATC	P_{chb} +9 SBS F
CKP 133	GTTTGTTAGTGTTTACTTACtttagctagTTATTTGGCCTTGT TTGATAATGGC	P_{chb} +9 SBS R
BBC 968	ATTATCAAACAAGTAAGTAAACACTAACAAACggccaaataa CTAGCTAAACCGTACCCG	P_{chb} -10 SBS F
BBC 967	TTAGCTAGttatttggccGTTTGTTAGTGTTTACTTACTTGTTT GATAATGGCTCTTTGC	P_{chb} -10 SBS R
BBC 966	GCCAAATAActagctaaacGTAAGTAAACACTAACAAACCGTA CCCGTTTTGCGAGTTG	P_{chb} +10 SBS F
BBC 965	GGGTACGGTTTGTTAGTGTTTACTTACgtttagctagTTATTT GGCCTTGTTTGATAATG	P_{chb} +10 SBS R
CKP 128	GTAAGTAAACACTAACAAACaggccaaataaCTAGCTAAACCG TACCCG	P_{chb} -11 SBS F
CKP 127	ttatttggcctGTTTGTTAGTGTTTACTTACTGTTTGATAATGG CTCTTTGCC	P_{chb} -11 SBS R
CKP 130	ctagctaaaccGTAAGTAAACACTAACAAACGTACCCGTTTTG CGAGTTGAATC	P_{chb} +11 SBS F
CKP 129	GTTTGTTAGTGTTTACTTACggtttagctagTTATTTGGCCTTG TTTGATAATGGC	P_{chb} +11 SBS R
CKP 124	GTAAGTAAACACTAACAAACaaggccaaataaCTAGCTAAACC GTACCCG	P_{chb} -12 SBS F
CKP 123	ttatttggccttGTTTGTTAGTGTTTACTTACGTTTGATAATGGC TCTTTGCC	P_{chb} -12 SBS R
CKP 126	ctagctaaaccgGTAAGTAAACACTAACAAACTACCCGTTTTGC GAGTTGAATC	P_{chb} +12 SBS F

CKP 125	GTTTGTTAGTGTTTACTTACcggtttagctagTTATTTGGCCTT GTTTGATAATGGC	P_{chb} +12 SBS R
**Primers for Overexpression Constructs**		
BBC 772	caatttcacacaggatcccgggAGGAGGTaacgtaATGGCCGGCAAT AAAAAAATC	P_{tac}-SlmA F
BBC 773	tgtaggctggagctgcttcTCACTTAATCTGTGCACTTAGC	P_{tac}-SlmA R
BBC 1236	caatttcacacaggatcccgggAGGAGGTaacgtaATGTTTAGGTTC TATCGAAAACAA	P_{tac}-ChiS F
BBC 577	tgtaggctggagctgcttcTTATTCACTGGTCAGGAGTTTTTGC	P_{tac}-ChiS R
CKP 224	tgtaggctggagctgcttcTTAtttgtcatcgtcatccttataatc	P_{tac} ChiS FLAG tag R
CKP 225	tgtaggctggagctgcttcTCACTTtttgtcatcgtcatc	P_{tac} SlmA FLAG tag R
**Primers for Vector Cloning**		
CKP 046	tatatatacatATGGCCGGCAATAAAAAAAT	SlmA NdeI F
CKP 047	tatatataggatccTCACTTAATCTGTGCA	SlmA BamHI R
CKP 076	tatatagaattcaggaggtaacgtaATGGCCGGCAATAAAAAAATC	SlmA EcoRI F
**Primers for EMSA Probes**		
BBC 744	cagcttcgcgtcctcggtaCGCAAATATAACTCAGGCAAAG	P_{chb} SBS F
CKP 072	cccgggatcctgtgtgaaattgCTTTGGCAGGAGTAAGAAAACACC TAG	P_{chb} SBS R
BBC 928	CAAATATATCCTCCTCACTATTTTG	Ec SBS Repression Reporter F
BBC 929	AACATCACCATCTAATTCAACAAG	Ec SBS Repression Reporter R
ABD 009	TATATGCCTTTAGGCATTAACTGTACTTCCGTC	P_{nanH} F
ABD 010	TGAAGTCATCTTGATTGACAAGTCTCCATCGAATG	P_{nanH} R
**Primers for qRT PCR**		
BBC 989	GCATCTAGGTTTTGACGTTTTTAACG	Uninduced transcript F
BBC 990	AACACTCTCCAAGACCTACCTC	Uninduced transcript R
BBC 918	AGTAATCGCAGCAGCAACCAG	Induced transcript F
BBC 919	GGTTCATAGATAAAGTCGGTGGTTG	Induced transcript R
ABD 132	CTGTCTCAAGCCGGTTACAA	*rpoB* F
ABD 133	TTTCTACCAGTGCAGAGATGC	*rpoB* R
**Primers for 5' RACE**		
BBC969	GATTACGCCAAGCTTCTGGTAACCCACTTGTTGATACCACG	GSP1 – for P_{chb} 5' RACE
BBC970	GATTACGCCAAGCTTAGCAGGCCGAAAGAGTAAACCACG	NGSP1 – for P_{chb} 5' RACE
**Primers for FLAG Tag Fusions**		
CKP 220	ggtgactacaaggatcacgacattgattataaggatgacgatgacaaaAAGT GAGTACAATGAGTCAAGATAAATACA	SlmA-3x FLAG F
CKP 221	ataatcaatgtcgtgatccttgtagtcaccatcatggtctttataatcAATCTGT GCACTTAGCAGCG	SlmA-3x FLAG R
CKP 237	GTGAAgattataaggatgacgatgacaaaTAAAGCAATCCGCAAGC GAG	ChiS-1x FLAG F
CKP 238	CTTTAtttgtcatcgtcatccttataatcTTCACTGGTCAGGAGTTTT TG	ChiS-1x FLAG R
